# Time interval between hCG administration and oocyte retrieval and ART outcomes: an updated systematic review and meta-analysis

**DOI:** 10.1186/s12958-023-01110-9

**Published:** 2023-07-03

**Authors:** Runxin Gan, Xi Huang, Jing Zhao, Qiong Zhang, Chuan Huang, Yanping Li

**Affiliations:** 1 Reproductive Medicine Center, Xiangya Hospital of Central South University, 87 Xiangya Road, Changsha, 410008 Hunan P.R. China; 2 Clinical Research Center for Women’s, Reproductive Health in Hunan Province, 87 Xiangya Road, Changsha, 410008 Hunan P.R. China; 3 Reproductive and Genetic Hospital of CITIC-Xiangya, Changsha, 410000 China

**Keywords:** Human chorionic gonadotropin, Oocyte retrieval, Ovulation, Meta-analysis, Interval, Assisted reproductive technology

## Abstract

**Research question:**

To explore whether prolonged hCG-ovum pickup interval improves assisted reproductive technology outcomes.

**Design:**

CENTRAL, CNKI, Cochrane Systematic Reviews, EMBASE, MEDLINE, PUBMED, and Web of Science up to May 13 2023 were searched for studies reporting associations between hCG-ovum pickup intervals and assisted reproductive technology outcomes. Intervention types included short (≤ 36 h) and long (> 36 h) hCG-ovum pickup intervals in assisted reproductive technology cycles. All outcomes were based upon only fresh embryo transfers. Primary outcome is defined as the clinical pregnancy rate. Data were pooled using random-effects models. Heterogeneity was assessed using the *I* 2 statistics.

**Results:**

Twelve studies were included in the meta-analysis, including five retrospective cohort studies, one prospective cohort study, and six randomized or quasi-randomized controlled trials. The short and long interval groups had similar oocyte maturation rates, fertilization rate and high-quality embryo rate (OR, 0.69; 95% CI, 0.45–1.06; *I* 2 = 91.1%, OR, 0.88; 95% CI, 0.77–1.0; *I* 2 = 44.4% and OR, 1.05; 95% CI, 0.95–1.17; *I* 2 = 8.6%, respectively). The clinical pregnancy rates in the long retrieval group were significantly higher than in the short retrieval group (OR, 0.66; 95% CI, 0.45–0.95; *I* 2 = 35.4%). The groups had similar miscarriage and live birth rates (OR, 1.92; 95% CI, 0.66–5.60; *I* 2 = 0.0% and OR, 0.50; 95% CI, 0.24–1.04; *I* 2 = 0.0%, respectively).

**Conclusions:**

The clinical pregnancy rates can be increased by prolonging the hCG-ovum pickup interval, which would help us develop more reasonable time schedules for fertility centers and patients.

**Meta-analysis registration:**

PROSPERO CRD42022310006 (28 Apr 2022).

**Supplementary Information:**

The online version contains supplementary material available at 10.1186/s12958-023-01110-9.

## Introduction

In assisted reproductive technology (ART), controlled ovarian stimulation (COS) aims to induce the growth of multiple dominant follicles and mature oocytes to improve the conception probability. During this process, the oocytes undergo a series of maturation steps—both nuclear and cytoplasmic—to attain a high fertilizing potential and developmental competence [1]. Two specific time points are crucially important in COS: (i) the ovulation triggering timing; (ii) the oocyte retrieval timing. In vivo oocyte maturation, which occurs during the interval between these two time points, is modulated by a complex series of biochemical events [2].

In a normal menstrual cycle, ovulation is triggered by a surge in luteinizing hormone (LH), whereas in COS cycles, this endogenous LH peak is mimicked by exogenous human chorionic gonadotropin (hCG). The LH surge or the hCG injected to induction ovulation promotes the activation of multiple signaling networks in the ovarian follicle, leading to the final steps of meiosis and oocyte maturation. These include synthesis of estrogen and prostaglandin, resumption of oocyte meiosis, cumulus expansion, reduction of cumulus cell-oocyte coupling, and synthesis of preovulatory enzymes [3]. Ovum pickup (OPU) is planned for a fixed time after hCG injection. Some studies have shown that the hCG-OPU interval considerably affects ART success. This interval is vital as some indispensable processes, including luteinization initiation, cumulus cell expansion, and oocyte meiosis resumption, should be well-established before aspiration [4]. It has been shown that, in addition to maternal age, the hCG-OPU interval was a significant factor associated with euploidy probabilities [5]. Therefore, this interval should be carefully managed to obtain the maximum number of eligible mature oocytes while avoiding spontaneous ovulation.

Physiology studies showed that ovulation occurs between 24 and 56 h after the LH surge onset, with a mean time of 32 h [6]. Based on hCG pharmacokinetics and its relationship with ovulation, Nader and Berkowitz [7] concluded that ovulation might occur earlier than 36 h in some women. They suggested aiming for a < 35 h interval if ovulation is to be avoided. Nevertheless, other studies had shown that ideal ART outcomes could be obtained when oocyte retrieval was done 36–39 h after hCG priming [4, 8, 9]. A meta-analysis of five randomized controlled trials (RCTs) from 2011 [10] concluded that compared to a shorter interval, an interval of > 36 h resulted in a higher fully expanded cumulus complex incidence and, consequently, a higher percentage of mature oocytes. However, it was also shown that the hCG-OPU interval was not associated with the oocyte retrieval rate [11]. The meta-analysis also shown that the prolonged interval did not increase the fertilization, implantation, and pregnancy rates. On the contrary, a study concluded that the prolonged ovulation trigger–OPU time interval in the long agonist protocol brings higher live birth rate (LBR) [12]. To date, there is no consensus regarding the optimal hCG-OPU interval, with a profound variance and intervals ranging between 32 and 38 h reported in clinical practice [9, 13]. Moreover, adhering precise interval plan on a regular basis, especially for a large ART center, is difficult. With ART development over the past several decades, various approaches to improve outcomes have been developed. More meta-analysis and systematic reviews are needed to assess the results of these developments. We performed an updated meta-analysis and systematic review based on the previous meta-analysis [10] to address the hCG-OPU interval question, which would help us develop more reasonable time schedules for fertility centers and patients, and support the search for better clinical outcomes.

## Methods

### Literature search

We registered this study in the International Prospective Register of Systematic Reviews (PROSPERO) with registration number CRD42022310006 on 28 Apr 2022. The study followed the Preferred Reporting Items for Systematic Review and Meta-Analysis (PRISMA) [14, 15]. Briefly, we searched CENTRAL, CNKI, Cochrane Systematic Reviews, EMBASE, MEDLINE, PUBMED, and Web of Science databases using key words that combined MeSH (Medical Subject Headings) terms and free text from database inception. We used the following main search terms: “Retrieval, Oocyte OR Oocyte Retrieval OR Oocyte Collection OR Collection, Oocyte OR Oocyte Aspiration OR Aspiration, Oocyte AND interval AND Gonadotropin, Chorionic OR Choriogonadotropin OR Choriogonin OR Pregnyl OR Chorulon OR Gonabion OR Biogonadil OR Chorionic Gonadotropin, Human OR Gonadotropin, Human Chorionic OR Human Chorionic Gonadotropin OR hCG (Human Chorionic Gonadotropin) OR Chorionic Gonadotropin.” The last search update was performed on May 13 2023. No language restriction was enacted, but the search was limited to studies in humans.

### Eligibility criteria, information sources, search strategy

All studies reporting associations between hCG-OPU intervals and ART outcomes were considered eligible for abstract screening. The study included infertility patients who underwent ART treatment cycles due to indications such as primary, tubal factor, male factor, or unexplained infertility and reported the outcomes. The COS protocols performed combined gonadotropin-releasing hormone agonist (GnRH-a) or antagonist (GnRH-A) or clomiphene and human menopausal gonadotropin (hMG) or follicle-stimulating hormone (FSH) or both. We evaluated the data eligibility in each study based on the following major exclusion criteria: (1) hCG was added to the culture medium rather than injected into the patients; (2) studies without clearly-defined outcome data; (3) abstract, reviews, letters, commentaries, case reports, and editorials; (4) insufficient data reported (i.e., impossible to extract primary data). If multiple published reports from the same study were available, only the one with the most detailed information for the primary data and outcome was included.

GRX and HX selected studies based on the established inclusion and exclusion criteria. The retrieved studies were screened for eligibility based on the title, abstract, and content. Disagreements on study eligibility were resolved by discussing with a third author (ZJ).

The ART program outcome measures based upon only fresh embryo transfers in the stimulation cycle were: primary outcome: (1) clinical pregnancy rate, calculated based on ultrasonographic visualization of a viable gestational sac in the uterine cavity after embryo transfer; Secondary outcome: (2) oocyte maturation rate, defined as the percentage of mature metaphase II (MII) oocytes, oocytes with the first polar body and a round ooplasm; (3) fertilization rate, defined as the number of two-pronuclear (2PN) zygotes divided by the number of aspirated MII oocytes; (4) high-quality embryo rate, defined as the number of embryos in which all blastomeres were of equal size without fragmentation, or blastomeres with unequal or equal size, with a maximum of 25% fragments of the embryo volume divided by the number of transferable embryos. (5) The live birth rate is defined as the number of live birth cycles divided by the number of transferred cycles.

### Intervention types

Maintaining a precise 36-h hCG-OPU interval is challenging in most IVF centers. Therefore, the commonly practiced interval is 32–38 h. Nevertheless, there is still no consensus nor a conclusive recommendation regarding the optimal hCG-OPU interval. The interval in the short interval group in the included studies of current meta-analysis was 33 to 36 h, and it was more than 36 to 41 h in the long interval group. Therefore, we used 36 h as the cutoff value between short and long intervals in the ART treatment cycles.

### Data extraction

GRX and HX extracted the baseline clinical data, procedure-specific data, and demographic characteristics from all included studies using a data extraction form. Disagreements were resolved by discussion. We recorded the following information from each study: first author, year of publication, study location, study type, sample size, patients’ characteristics (e.g., mean age), infertility duration, infertility causes, hCG usage and dose, ART methods, and oocyte maturation, fertilization, high-quality embryo, clinical pregnancy, miscarriage, and live birth rates.

### Assessment of risk of bias

The studies’ quality of RCTs and cohort studies was assessed using the Cochrane Risk of Bias 2 (RoB 2) tool [16] and the Newcastle–Ottawa scale respectively (NOS) [17]. RoB2 encompasses five aspects: study eligibility criteria, identification of relevant information, bias domains, risk of bias assessments, judgments and overall risk of bias, and presentation of results. The scale of NOS consists of nine items covering three dimensions: (1) selection, with a maximum score of 4; (2) comparability, with a maximum score of 2; (3) exposure (case–control)/outcome (cohort), with a maximum score of 3. The maximum possible score is nine, indicating a high-quality study. ZJ and ZQ independently scored all studies. Disagreements were resolved by discussion.

### Data synthesis

We calculated the summary estimates to determine the oocyte maturation, fertilization, clinical pregnancy, miscarriage, and live birth rates for this systematic review and meta-analysis. The data were analyzed using the Stata software (Version 16.0; Stata Corporation, College Station, TX). Results for dichotomous variables are expressed as odds ratios (ORs) and 95% confidence intervals (CIs). We used random-effects models to calculate the summary estimates and their 95% CIs. For the total or subtotal of each variable, we report the χ^2^-test statistic for heterogeneity across studies with its degree of freedom and *P*-value, the *I* ^2^ statistic that measured the extent of inconsistency in the results, and the *Z* statistic with its *P*-value for the overall effect. *I* ^2^ values above 50% suggested considerable heterogeneity, 25–50% suggested modest heterogeneity, and values below 25% indicated low heterogeneity. These estimates can have marked uncertainty, especially when only a few trials are assessed, and should be interpreted cautiously. We conducted sensitivity analysis by removing one study at a time and repeating the analysis to assess the consistency and quality of our meta-analysis results. The Egger’s test was used to check for publication bias.

## Results

### Study selection

**Fig. 1:**
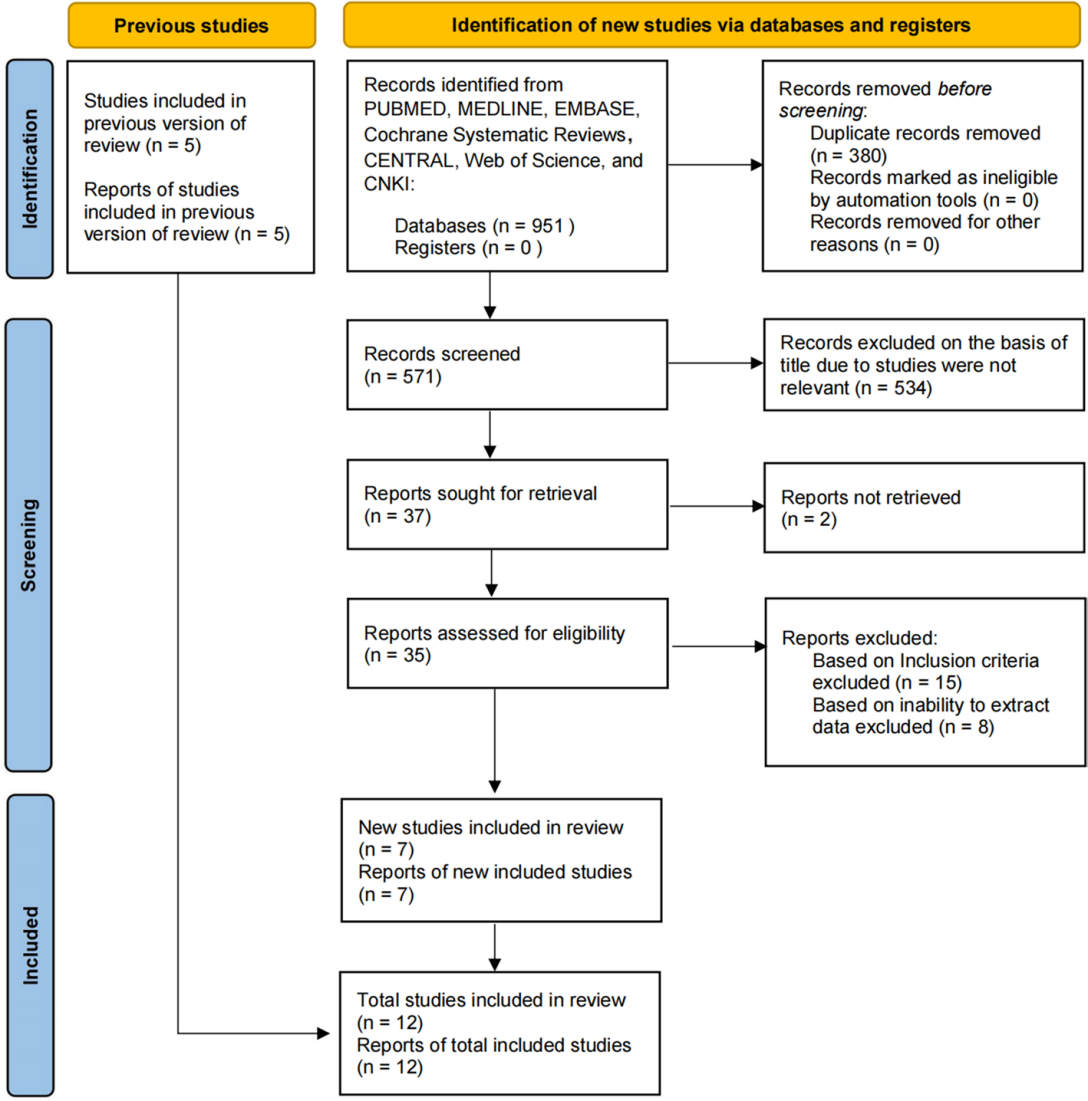
Flow diagram of search strategy

We retrieved 951 relevant articles. After removing duplicates and screening the titles, 37 articles remained. Two articles were excluded after reading the abstracts. After reviewing the full text of the remaining 35 studies, 15 were excluded. eight of the remaining 20 studies were excluded as it was impossible to extract primary data from them. Finally, 12 studies were included in this meta-analysis, nine in English and three in Chinese (Fig. 1) [9, 13, 18–27]. The study methodology assessment on the Newcastle–Ottawa Scale is presented in Table 1. The studies included five retrospective cohort studies, one prospective cohort study, and six randomized or quasi-randomized controlled trials.

**Table 1:** Quality assessment of each eligible study according to the NOS

Study	Selection				Comparability 1	Outcome			Scores
	1	2	3	4		1	2	3	
Skvirsky S (2021) [27]	*	*	*	*	*	*	*	*	8
Bao L (2019) [26]	*	*	*	*	*	*	*	*	8
Chen YY (2014) [25]	*	*	*	*	*	*		*	7
Ghasemian F (2013) [24]	*	*	*	*		*		*	6
Gong SQ (2012) [23]	*	*	*	*	*	*	*	*	8
Bokal EV (2005) [21]	*	*	*	*		*		*	6

*NOS* Newcastle–Ottawa Quality Assessment Scale

### Study characteristics

The retrieved studies were done in Israel, Australia, England, Norway, Egypt, Slovenia, China, and Scotland between 1990 and 2021. The ART outcomes based upon only fresh embryo transfers in the stimulation cycle reported included oocyte maturation rate in five studies, fertilization rate in eight, high-quality embryo rate in six, clinical pregnancy rate in nine, miscarriage rate in three, and live birth rate in two. One patient from 37 h group in the twelve included studies encountered spontaneous ovulation [20]. The characteristics of included studies are summarized in Tables 2 and 3.

**Table 2:** Main characteristics of studies included in the meta-analysis

Author	Location of study	Study type	Sample size (SI/LI)	Mean age (years)	controlled ovarian stimulation protocol (s)	ART	Duration of infertility (years)	Causes of infertility	Dose of hCG
Skvirsky S et al. (2021) [27]	Israel	Retrospective cohort study	485(482/3)	SI: 34.9 ± 5.6 LI: 35.8 ± 8.0	GnRH agonist/ GnRH antagonist	IVF/ICSI	NA	NA	250 μg, subcutaneously
Bao L et al. (2019) [26]^a^	China	Retrospective cohort study	199(78/121)	SI: 28.95 ± 3.09 LI: 28.89 ± 3.43	GnRH agonist	IVF/ICSI	SI:3.82 ± 1.86 LI:3.74 ± 2.10	NA	250 μg, subcutaneously
Chen YY et al. (2014) [25]^a^	China	Retrospective cohort study	1500(1337/163)	SI: NA LI: 30.232 ± 3.879	GnRH agonist	IVF/ICSI	NA	NA	5 000 ~ 10 000 IU, IM
Ghasemian F et al. (2013) [24]	Iran	Retrospective cohort study	126(65/61)	NA	GnRH agonist	ICSI	NA	NA	10,000 IU, NA
Gong SQ et al. (2012) [23]^a^	China	Retrospective cohort study	173(41/132)	SI: 33.93 ± 3.44 LI: 31.22 ± 3.70	GnRH agonist	IVF/ICSI	SI:4.31 ± 2.87 LI:4.11 ± 2.43	NA	6 000 ~ 10 000 IU, IM
Raziel A et al. (2006) [22]	Israel	Quasi-randomized trial study	72(36/36)	31.3 ± 4.9	GnRH agonist	ICSI	5 ± 3.6	Primary infertility	5000 IU, IM
Bokal EV et al. (2005) [21]	Slovenia	Prospective cohort study	20(9/11)	30.9 ± 3.1	GnRH agonist	IVF/ICSI	NA	NA	10,000 IU, NA
Nargund G et al. (2001) [13]	England	Randomized study	369 (258/111)	33.4	GnRH agonist	IVF	NA	Tubal damage, male factor, and unexplained infertility	10,000 IU, NA
Bjercke S et al. (2000) [20]	Norway	Randomized study	170 (83/87)	SI: 35 LI: 33	GnRH agonist	IVF	NA	Tubal factor	10,000 IU, subcutaneously
Mansour RT et al. (1994) [9]	Egypt	Randomized study	90(60/30)	SI: NA LI: 31.2 ± 2.3	GnRH agonist	ICSI	NA	Male factor	10,000 IU, IM
Jamieson ME et al. (1991) [19]	Scotland	Randomized study	60 (30/30)	NA	GnRH agonist	IVF	NA	Tubal factor or unexplained infertility	5000 IU, NA
Thornton SJ et al. (1990) [18]	Australia	Quasi-Randomized study	214 (108/106)	NA	CC	IVF	NA	NA	5,000 IU, NA

^a^Chinese-language study

**Table 3:** Main characteristics of studies included in the meta-analysis

Author	Oocyte maturation rate	Fertilization rate	High-quality embryo rate	Clinical pregnancy rate	Miscarriage rate	Live birth rate
Skvirsky S et al. (2021) [27]	NA	NA	SI:9.1% (44 /481) LI:0% (0 /3)	SI: 29.7% (143/482) LI:33.3% (1/3)	NA	SI: 23.7% (114/482) LI:33.3% (1/3)
Bao L et al. (2019) [26]^a^	SI:93.05% (1031 /1108) LI:93.51% (1803 /1928)	SI:85.25% (879 /1031) LI:85.52% (1542 /1788)	SI:39.02% (343 /879) LI:39.23% (605 /1542)	SI:43.49% (20 /46) LI:62.29% (42 /67)	NA	SI:39.13% (18 /46) LI:56.71% (38 /67)
Chen YY et al. (2014) [25]^a^	SI:86.9% (12,678 /14586) LI:87.7% (1496 /1704)	SI:77% (8508 /11051) LI:80.8% (1014 /1255)	SI:72.4% (6049 /8358) LI:70.9% (708/999)	NA	NA	NA
Ghasemian F et al. (2013) [24]	NA	NA	SI:73.3% (356 /486) LI:66.8% (266/398)	SI:21.83% (5 /24) LI:24.51% (25/102)	NA	NA
Gong SQ et al. (2012) [23]^a^	NA	SI: 80.4% (258/321) LI:80.7% (1192 /1477)	SI: 45.8% (114/249) LI:45.6% (537 /1177)	SI:29.7% (11 /37) LI:40.3% (50/124)	SI:18.2% (2 /11) LI:8% (4/50)	NA
Raziel A et al. (2006) [22]	SI:50% (248 /497) LI:72% (434 /604)	SI: 64% (159/248) LI:67% (291 /434)	SI: 52% (52/100) LI:60% (60 /100)	SI:17% (6/36) LI:33% (12/36)	SI:11% (4 /36) LI:8% (3/36)	NA
Bokal EV et al. (2005) [21]	SI:83.5% (76 /91) LI:80.3% (94 /117)	SI:51.3% (39/76) LI:67% (63 /94)	NA	NA	NA	NA
Nargund G et al. (2001) [13]	NA	NA	NA	SI:10.5% (27/258) LI:18% (20/111)	NA	NA
Bjercke S et al. (2000) [20]	NA	SI:63.9% (554/867) LI:62.1% (598 /963)	NA	SI:24% (20/83) LI:20% (17/87)	NA	NA
Mansour RT et al. (1994) [9]	SI:64.7% (345/539) LI:79.47% (209/263)	SI:55.9% (193/345) LI:57.42% (120 /209)	NA	NA	NA	NA
Jamieson ME et al. (1991) [19]	NA	SI:76.8% (232/302) LI:84.2% (255 /303)	NA	SI:20% (6/30) LI:26.7% (8 /30)	NA	NA
Thornton SJ et al. (1990) [18]	NA	NA	NA	SI:18.5% (17/92) LI:14.4% (13 /90)	SI:17.6% (3 /17) LI:0% (0)	NA

^a^Chinese-language study

### Quality assessment of included studies and synthesis of results

The meta-analysis outcome measures’ results are presented in Table 4.

**Table 4:** Meta-analysis results of ART outcome measures

Outcome measures	No. Of studies	Statistical mode	Heterogeneity (I^2^,%)	Chi^2^	degree of freedom	*P* value	Effect size	95% CI	Z	*P* value
Oocyte maturation rate	5	Random	91.1	44.80	4	0.000	OR	0.69 (0.45–1.06)	1.67	0.094
Fertilization rate	8	Random	44.4	12.59	7	0.083	OR	0.88 (0.77—1.0)	2.00	0.045
High-quality embryo rate	6	Random	5.47	8.6	5	0.361	OR	1.05 (0.95–1.17)	1	0.316
Clinical pregnancy rate	9	Random	35.4	12.39	8	0.135	OR	0.66 (0.45–0.95)	2.21	0.027
Miscarriage rate	3	Random	0	0.32	2	0.851	OR	1.92 (0.66—5.60)	1.20	0.232
Live birth rate	2	Random	0	0.03	1	0.856	OR	0.50(0.24–1.04)	1.86	0.063

RoB 2 indicated high risk for 4 studies, some concerns for 2 studies for the physical function outcome (Supplementary Table SI and justifications in supplement Appendix 1). Overall, we judged the both primary outcome and secondary outcome as high risk of bias. Two study utilized odd or even IVF identity numbers of patients for patient allocation, this trial was known as quasi-randomized trial [18, 22]. There was only one trial [9] that mentioned blinding, where the gynecologist performing the oocyte aspiration was unaware of the time interval. Additionally, only one trial [20] provided a sufficient description of allocation concealment, with randomization carried out through the use of sealed envelopes drawn by a non-participating nurse. Both studies had no loss to follow-up, complete outcome data, and no selective reporting bias. Following the NOS criteria, all of the included cohort studies achieved the score ≥ 6, with the score ranging from 6 to 8 (Table 1).

**Fig. 2:**
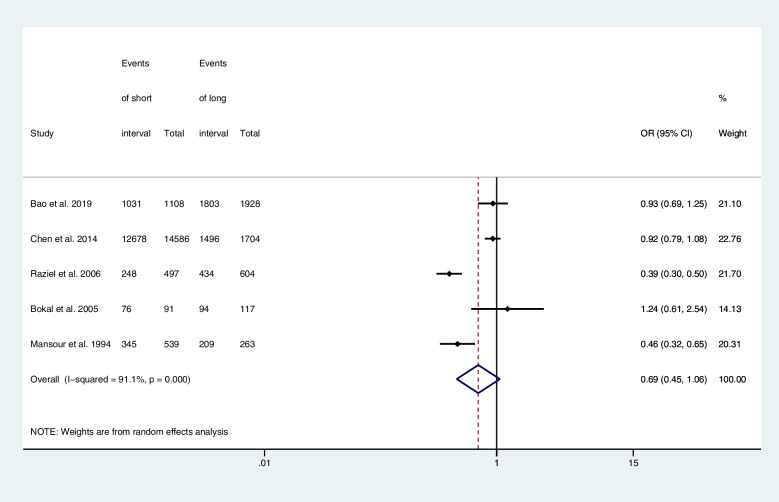
Forest plot of oocyte maturation rate of short interval versus long interval in ART program

Five articles provided computable data on oocyte maturation rates, including two RCTs [9, 22], two retrospective cohort studies [25, 26], and one prospective cohort study [21]. Figure 2 shows the ORs and 95% CIs of the association between the oocyte maturation rate and the hCG-OPU interval, covering 21,437 retrieved oocytes in five studies, of which 18,414 reached the MII stage. The short and long interval groups had similar oocyte maturation rates (OR, 0.69; 95% CI, 0.45–1.06; *I* ^2^ = 91.1%). The combined OR of the overall risk estimate was consistent and without apparent fluctuation.

**Fig. 3:**
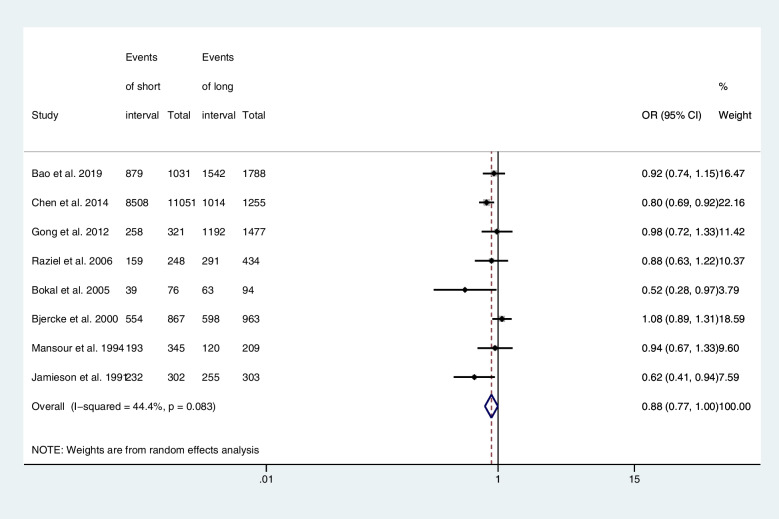
Forest plot of fertilization rate of short interval versus long interval

Eight articles provided computable data on fertilization rate, including four RCTs [20, 22], three retrospective cohort studies [23, 25, 26], and one prospective cohort study [21]. Figure 3 shows the ORs and 95% CIs of the association between the fertilization rate and the hCG-OPU interval, covering 20,764 MII oocytes retrieved in eight studies, of which 15,897 were fertilized to become 2PN zygotes. The short and long interval groups had similar fertilization rate (OR, 0.88; 95% CI, 0.77–1.0; *I* ^2^ = 44.4%).

**Fig. 4:**
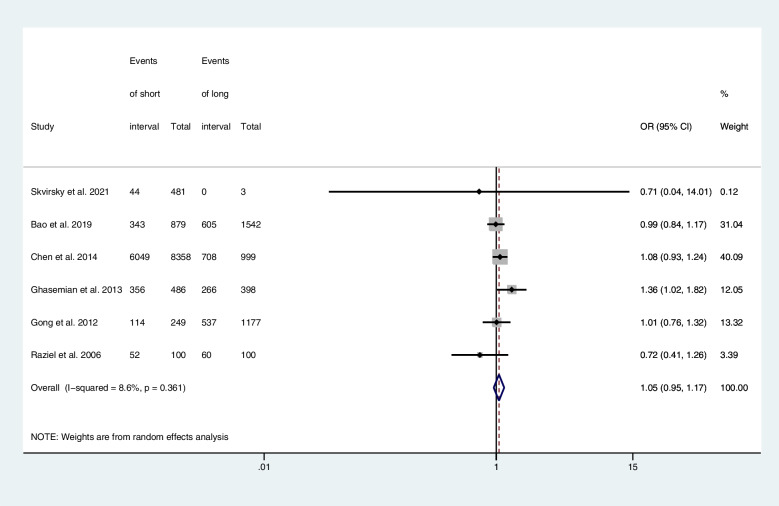
Forest plot of high-quality rate of short interval versus long interval in ART program

Six articles provided computable data on the high-quality embryo rate, including one RCTs [22], five retrospective cohort studies [23–26, 28]. Figure 4 shows the ORs and 95% CIs of the association between the high-quality embryo rate and the hCG-OPU interval, covering 14,772 embryos in two studies, of which 9134 were high-quality embryos. The high-quality embryo rate in the short interval group was higher than in the long interval group, although there is no statistical significance (OR, 1.05; 95% CI, 0.95–1.17; *I* ^2^ = 8.6%).

**Fig. 5:**
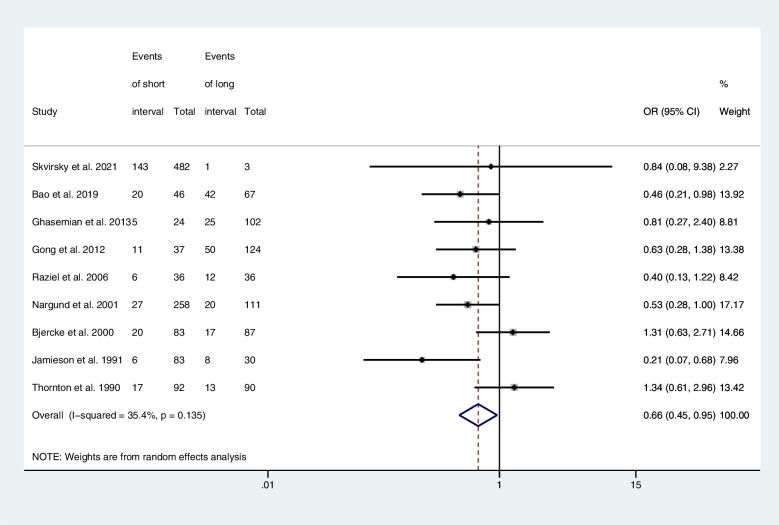
Forest plot of clinical pregnancy rate of short interval versus long interval in ART program

Nine articles provided computable data on clinical pregnancy rate, including five RCTs [13, 18–20, 22], four retrospective cohort studies [23, 24, 26, 27]. Figure 5 shows the ORs and 95% CIs of the association between the clinical pregnancy rate and the hCG-OPU interval, covering 1,791 patients in nine studies, of which 443 became pregnant after embryo transfer. The clinical pregnancy rate in the long interval group was significantly higher than in the short interval group (OR, 0.66; 95% CI, 0.45–0.95; *I* ^2^ = 35.4%).

**Fig. 6:**
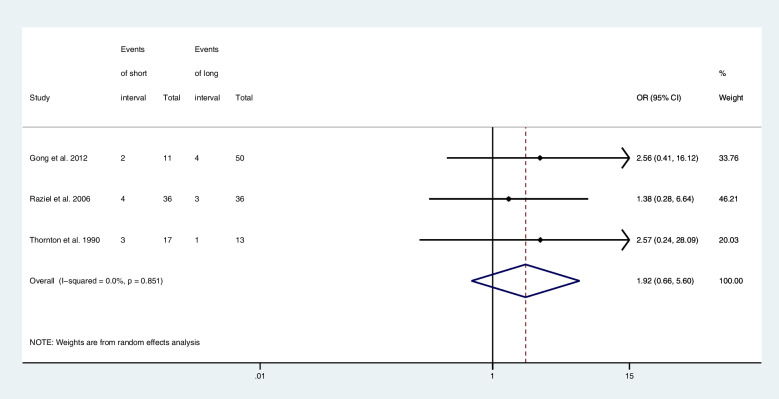
Forest plot of miscarriage rate of short interval versus long interval in ART program

Three articles provided computable data on miscarriage rate, including two RCTs [18, 22], one retrospective cohort studies [23]. Figure 6 shows the ORs and 95% CIs of the association between the miscarriage rate and the hCG-OPU interval, covering 163 patients in three studies, of which 17 experienced a miscarriage. The miscarriage rate in the short interval group was higher than in the long interval group, although there is no statistical significance (OR, 1.92; 95% CI, 0.66–5.60; *I* ^2^ = 0.0%).

**Fig. 7:**
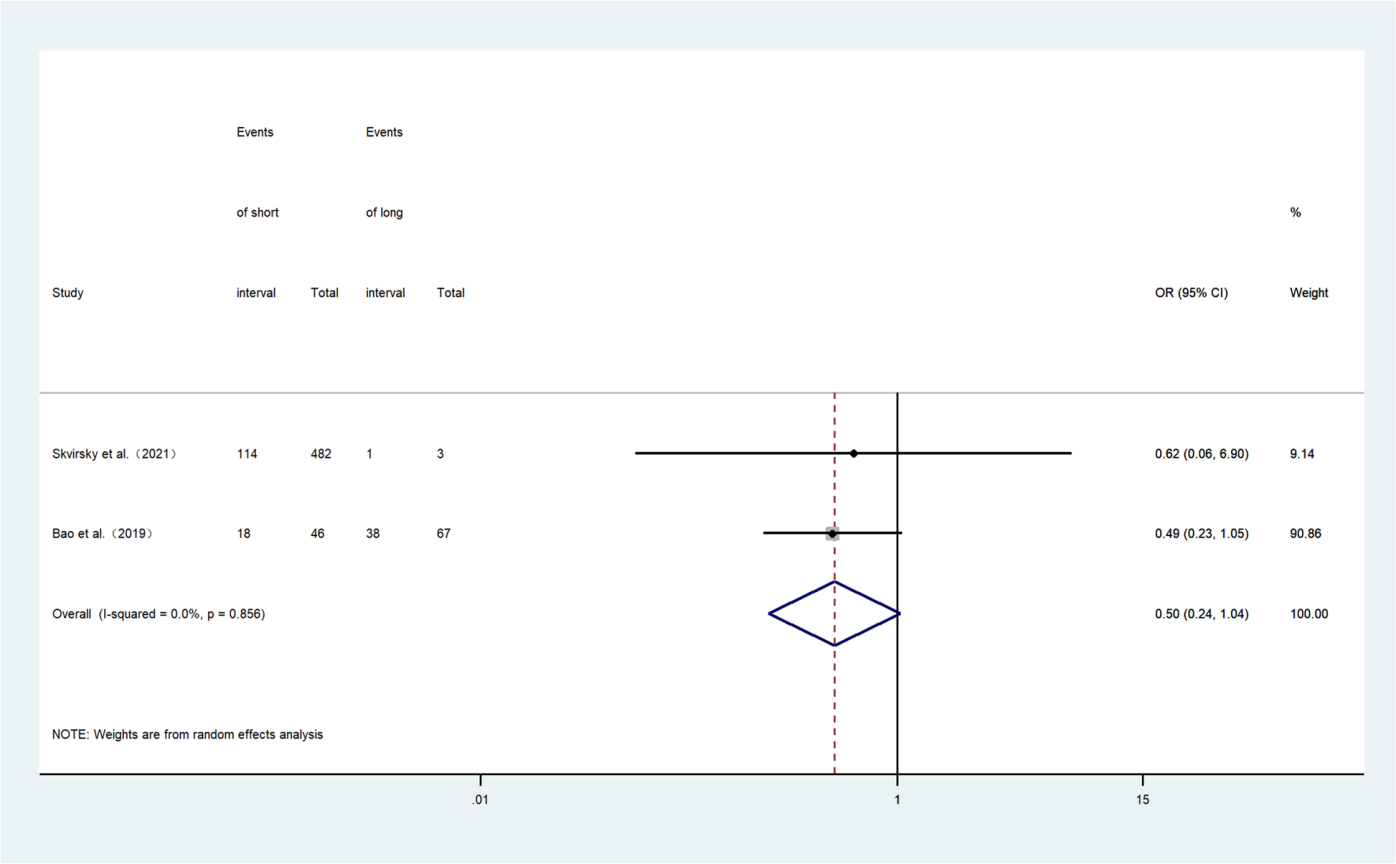
Forest plot of live birth rate of short interval versus long interval in ART program

Two retrospective cohort studies provided computable data on live birth rate [26, 27]. Figure 7 shows the ORs and 95% CIs of the association between the live birth rate and the hCG-OPU interval, covering 598 patients in two studies, of which 171 had a live birth. The live birth rate in the short interval group was lower than in the long interval group, although there is no statistical significance (OR, 0.50; 95% CI, 0.24–1.04; *I* ^2^ = 0.0%).

### Sensitivity analysis

We performed a sensitivity analysis for oocyte maturation, fertilization, clinical pregnancy, high-quality embryo and miscarriage rates (Supplementary Figures S1, S2, S3 S4 and S5) by removing one study at a time and assessing the effect on the pooled results. Since only two studies reported live birth rates, sensitivity analysis was not performed. The pooled results for the oocyte maturation, fertilization, clinical pregnancy and miscarriage rates were stable.

### Risk of bias of included studies

We performed a publication bias analysis for the oocyte maturation, fertilization, high-quality embryo and clinical pregnancy rates (Supplementary Figures S6, S7, S8 and S9), finding no evidence for publication bias. Publication bias analysis was not performed because only 3 and 2 studies reported miscarriage and live birth rates respectively.

## Discussion

Our results differed from the previous meta-analysis. We found no difference between the short and long interval groups in the oocyte maturation rate and fertilization rate, but the clinical pregnancy rates in the long interval group were significantly higher than in the short interval group. We also performed a meta-analysis of miscarriage and live birth rates, finding no difference between the groups. One patient from 37 h group in the twelve included studies encountered spontaneous ovulation [20]. Therefore, we think that in clinical practice, ART patients may be able to benefit from a moderately extended time interval between hCG and oocyte retrieval (36-38 h).

A wide variety of hCG doses were used in the included twelve studies, with the lowest being 5,000 IU. The hCG dose was determined according to the patients’ response to COS and their estradiol levels. Regardless of the route of administration, hCG at doses of 5,000 and 10,000 IU achieved similar oocyte maturation, fertilization, and pregnancy rates. Therefore, it was suggested that differences between hCG doses do not affect the ART outcomes [29, 30]. One study used a clomiphene citrate protocol for COS. Clomiphene citrate has an antiestrogenic effect and may suppress a premature LH surge. However, the inhibition function of clomiphene citrate is less effective than GnRH analogs, as suggested by the higher cancelation rate [31]. Therefore, spontaneous ovulation may have occurred in one patient in the included study that used the clomiphene citrate protocol.

The previous meta-analysis [10] included five RCTs with 895 participants and showed that the oocyte maturation rate in the long interval group (> 36 h) was higher than in the short interval group (< 36 h). However, two of the five included studies were from ICSI cycles. One of the included studies [9] found that the percentage of MII oocytes increased from 49.6% to 77.4% and 79.4% when the hCG-OPU interval was extended from 35 to 36 and 37 h, respectively (*P* < 0.001). The main disadvantage of that study was its limited sample size of only 30 patients per group. Several other studies have reached similar conclusions [24, 32, 33]. On the contrary, the results of our meta-analysis found similar maturation rates in the short and long interval groups (85.6 and 87.4%, respectively). Some other studies also reported no difference in oocyte maturation rates between various interval groups, and a linear regression analysis found no correlation between the oocyte maturation rate and the hCG-OPU interval length [28, 34, 35]. Our meta-analysis found that two of the five studies reporting oocyte maturation rates included patients with polycystic ovary syndrome (PCOS) [21, 26]. One study found that antral follicle count was negatively associated with the rate of matured oocytes [12]. Those authors mentioned that although a higher antral follicle count could lead to more retrieved oocytes, it also contributed to a higher proportion of small immature oocytes on the retrieval day. Geng et al. [36] observed that 21% of the patients with PCOS showed premature luteinization before follicle maturation. Furthermore, when ICSI is performed, the cumulus cells are removed allowing direct assessment of the oocyte on the day of retrieval. With conventional insemination, cumulus cells are typically not removed initially, and oocyte maturity is typically assessed the following day. These could lead to the lack of difference in oocyte maturation rate between the two groups. Regarding the fertilization rate, our results were the same as those of the previous meta-analysis that the short and long interval groups had similar fertilization rate. We find, quite interestingly, high-quality embryo rate in the short interval group was higher than in the long interval group, although there is no statistical significance. Furthermore, the results of our meta-analysis also revealed that the clinical pregnancy rates in the long interval group were significantly higher than in the short interval group. More specifically, a recent retrospective cohort study provided some degree of support for our findings, indicating that there is a significant positive correlation between the oocyte retrieval period and the clinical pregnancy occurrence up to the 37th hour, where retrieval at the 37th hour was found to provide the most optimal outcome [37]. Although there is a difference in clinical pregnancy rates between the two groups, there is no statistically significant difference in miscarriage rates and live birth rates. In our study, a total of 9 articles were included, comprising 1791 participants, for the comparison of clinical pregnancy rates. Only 2 articles, with a total sample size of 598, were included for the comparison of live birth rates, and 3 articles, with a total sample size of 163, were included for the comparison of miscarriage rates. When pooling the data for the comparison of miscarriage rates and live birth rates, the overall miscarriage rate was lower in the long interval group compared to the short interval group, while the overall live birth rate was higher in the long interval group compared to the short interval group. This may be attributed to the limited number of included articles and the small sample sizes, which might not have reached statistical significance. Furthermore, the interval from hCG injection to oocyte injection in ICSI cycles might impact the outcomes. Adjusted analysis in the study by Vandenberghe et al. [38] also showed that oocytes injected less than 36 h after triggering ovulation had insignificantly lower live birth rate than oocytes injected 38 h after triggering ovulation (OR, 0.533; 95% CI, 0.252–1.126; *P* = 0.099). We included studies with ICSI cycles in our analysis, and we do not know how long the hCG-to-oocyte injection interval was in these studies, potentially affecting our results. Future studies will also need to focus on the hCG-ICSI interval.

As is well known, oocyte maturation involves nuclear and cytoplasmic maturation [39]. Nuclear maturation includes recovery from the first meiosis, germinal vesicle breakdown, and first polar body formation. Unlike the nucleus, there are no clear criteria to define or detect cytoplasmic maturation, an extremely complex process. During natural maturation, the cytoplasm and nucleus might mature in a synchronized way, which might not be the case in induced ovulation cycles [40]. Oocyte maturation is a continuous process that does not stop at oocyte retrieval. The in vitro and in vivo maturation processes contribute equally to the final oocyte maturation assessment. Therefore, the maturation assessment timing is more vital than the hCG injection and oocyte retrieval timings. Consequently, we hypothesized that prolonging the hCG-OPU interval might benefit the oocyte cytoplasmic maturation or enhance ovum competence, thereby improving the clinical pregnancy rates. Several studies agree with our hypothesis [32, 41, 42]. Furthermore, oocyte cytoplasmic immaturity was associated with metaphase plate anomalies and aneuploidies [43]. It was also observed that the euploidy rate was highest in the 38–39 h interval and lowest in the 34–35 h interval [5]. Longer hCG-OPU interval increases the production of oocytes with fully expanded cumulus, which might reflect oocyte maturation. Researchers presumed that a longer interval increases the proportion of oocytes heading for fertilization and cleavage, implying that long intervals might improve gamete quality by allowing the more optimal in vivo maturation process to continue. Bokal et al. [21] found that prolonging the hCG-OPU interval in patients with PCOS improved the expression and action of angiogenic substances such as those of the renin-angiotensin system and vascular endothelial growth factor, enhancing follicular vascularization and, consequently, improving oocyte quality, fertilization competence, and embryo developmental potential. Besides, many factors are involved in oocyte maturation and embryo development, including angiotensin II, vascular endothelial growth factor, interleukin (IL)-1, IL-6, IL-8, angiopoietin, insulin-like growth factor, basic fibroblast growth factor, and endothelin. These have a time-dependent effect after hCG priming [21, 44, 45].

Apart from the necessary hCG-OPU interval required for oocyte maturation, we need to consider the risk of spontaneous ovulation. Templeton et al. [46] reported that spontaneous ovulation occurred in five patients: one in the no-hCG injection group, one in the 24-h group, and three in the 36-h group. Andersen et al. [47] found a mean interval of 38.3 h from hCG injection to first follicular rupture. Fleming and Coutts [8] reported that no ovulation occurred within an interval of fewer than 39.5 h. Gudmundsson et al. [4] found that 39 h was a critical cutoff to avoid spontaneous ovulation. Nargund et al. [13] found no case of spontaneous ovulation even though the longest interval was 41 h. One patient from 38 h group in the twelve included studies encountered spontaneous ovulation [20]. Therefore, the interval should be carefully controlled to ensure an optimal maturation rate while avoiding spontaneous ovulation that leads to treatment cycle cancellation.

This systematic review has several strengths. Unlike the previous meta-analysis [10] that included only randomized and quasi-randomized controlled trials, we did not limit the study type and included RCTs and cohort studies in this meta-analysis. And we performed a sensitivity and publication bias analysis. The 12 selected studies for this meta-analysis included a large amount of data. Our secondary outcomes increased the high-quality embryo rate, miscarriage and live birth rates, although data were available for only 3 and 2 studies regarding miscarriage and live birth rates, respectively. Our findings would help develop more reasonable time schedules for fertility centers and patients, and support the search for better clinical outcomes.

The interval groups were selected based on clinical relevance rather than to ensure a homogenous division suitable for the statistical analysis. Despite our efforts to contact the authors and co-authors of some relevant studies lacking detailed data, we encountered difficulties in obtaining detailed data from other studies. The ART outcomes were associated with factors such as the patient’s age, endocrine profile, endometrial receptivity, and cross-talk between the embryo and the endometrium, in addition to the hCG-OPU interval. However, the absence of detailed comparative information on patient characteristics such as age and endocrine profile in the included studies could lead to a bias, which is one of our study’s limitations. we encountered difficulties in obtaining detailed data from other research studies despite our efforts to reach out to the respective authors and co-authors. With conventional insemination, cumulus cells are typically not removed initially, and oocyte maturity is typically assessed the following day. When ICSI is performed, the cumulus cells are removed allowing direct assessment of the oocyte on the day of retrieval. This could introduce considerable bias. There was high heterogeneity of results and some of the retrospective studies included in this meta-analysis were of moderate quality. We performed sensitivity analysis, and publication bias to minimize the effect. Overall, our conclusion will need to be validated in future RCTs. Moreover, the stimulation protocol might affect the optimal hCG-OPU interval duration [12] and increase study bias. Since only two studies included clomiphene citrate and a GnRH antagonist, subgroup analysis could not be performed.

Based on the previous meta-analysis, current updated meta-analysis further demonstrates the benefit of appropriately extended hCG-OPU interval for patients in IVF-ET treatment cycle. Our findings encourage conducting future large, well-powered, multicenter RCTs in various countries and regions to explore the question of hCG-OPU interval and euploidy. The outcomes of included studies were based upon only fresh embryo transfers in the stimulation cycle and we could not compare cumulative pregnancy rate and live birth rate. We recommend that these studies use a predefined and consistent set of clinically meaningful definitions and internationally-agreed outcomes including cumulative pregnancy rate and live birth rate. We anticipate that the effect the hCG-OPU interval has on the ART outcomes would vary among patients, COS protocols, and even fertilization methods, making it an important unanswered clinical question. Another important question is how long should the hCG-OPU interval be to help obtain optimal ART outcomes while avoiding spontaneous ovulation? Moving forward, a clear, evidence-based strategy for prolonging the hCG-OPU interval in women undergoing IVF therapy is required.

## Conclusion

The clinical pregnancy rates can be increased by moderately prolonging the hCG-ovum pickup interval. Similar oocyte maturation was noted in the short and long interval groups. Regarding the high-quality embryo, miscarriage and live birth rates, the long interval group were better than the short interval group, although there were no statistically significances. which would help us develop more reasonable time schedules for fertility centers and patients. In view of the above, the clinical pregnancy rates can be increased by prolonging the hCG-ovum pickup interval, which would help us develop more reasonable time schedules for fertility centers and patients.

## Supplementary Information

**Additional file 1: Figure S1.** Meta-analysis, sensitivity analysis, and random-effects estimates examining the oocyte maturation rate of short interval versus long interval in ART program.

**Additional file 2: Figure S2.** Meta-analysis, sensitivity analysis, and random-effects estimates examining the fertilization rate of short interval versus long interval in ART program.

**Additional file 3: Figure S3.** Meta-analysis, sensitivity analysis, and random-effects estimates examining the high-quality rate of short interval versus long interval in ART program.

**Additional file 4: Figure S4.** Meta-analysis, sensitivity analysis, and random-effects estimates examining the clinical pregnancy rate of short interval versus long interval in ART program.

**Additional file 5: Figure S5.** Meta-analysis, sensitivity analysis, and random-effects estimates examining the miscarriage rate of short interval versus long interval in ART program.

**Additional file 6: Figure S6.** Publication bias analysis of oocyte maturation rate of short interval versus long interval in ART program.

**Additional file 7: Figure S7.** Publication bias analysis of fertilization rate of short interval versus long interval in ART program.

**Additional file 8: Figure S8.** Publication bias analysis of high-quality rate of short interval versus long interval in ART program.

**Additional file 9: Figure S9.** Publication bias analysis of clinical pregnancy rate of short interval versus long interval in ART program.

**Additional file 10: Table SIa.** ROB for oocyte maturation outcome. **Table SIb.** ROB for fertilization outcome. **Table SIc.** ROB for high-quality embryo outcome. **Table SId.** ROB for clinical pregnancy outcome. **Table SIe.** ROB for miscarriage outcome.

**Additional file 11: Appendix 1.** Justifications in ROB.

## Data Availability

This meta-analysis was based on the data from the published articles and independent of any patient involvement. All the data will be made available to the editors of the journal for review or query upon.

## Abbreviations

hcg: Human chorionic gonadotropin
ART: Assisted reproductive technology
COS: Controlled ovarian stimulation
LH: Luteinizing hormone
OPU: Ovum pickup
LBR: Live birth rate
hMG: Human menopausal gonadotropin
FSH: Follicle-stimulating hormone
GnRH-a: Gonadotropin-releasing hormone agonist
GnRH-A: Gonadotropin-releasing hormone antagonist
MII: Mature metaphase II
2PN: Two-pronuclear
OR: Odds ratio

## References

[1] Watson AJ (2007). Oocyte cytoplasmic maturation: a key mediator of oocyte and embryo developmental competence. J Anim Sci.

[2] Son WY, Lee SY, Lim JH (2005). Fertilization, cleavage and blastocyst development according to the maturation timing of oocytes in in vitro maturation cycles. Hum Reprod.

[3] Benyo DF, Ravindranath N, Bassett S, Hutchison J, Zeleznik AJ (1993). Cellular aspects of corpus luteum function in the primate. Hum Reprod.

[4] Gudmundsson J, Fleming R, Jamieson ME, McQueen D, Coutts JR (1990). Luteinization to oocyte retrieval delay in women in whom multiple follicular growth was induced as part of an in vitro fertilization/gamete intrafallopian transfer program. Fertil Steril.

[5] Lee CI, Chen HH, Huang CC, Chen CH, Cheng EH, Huang JY, Lee MS, Lee TH (2020). Effect of Interval between Human Chorionic Gonadotropin Priming and Ovum Pick-up on the Euploid Probabilities of Blastocyst. J Clin Med.

[6] World Health Organization TFoMftDotFP, Special Programme of Research, Development and Research Training in Human Reproduction (1980). Temporal relationships between ovulation and defined changes in the concentration of plasma estradiol-17 beta, luteinizing hormone, follicle-stimulating hormone, and progesterone. I. Probit analysis. World Health Organization, Task Force on Methods for the Determination of the Fertile Period, Special Programme of Research, Development and Research Training in Human Reproduction. Am J Obstet Gynecol.

[7] Nader S, Berkowitz AS (1990). Study of the pharmacokinetics of human chorionic gonadotropin and its relation to ovulation. J In Vitro Fert Embryo Transf.

[8] Fleming R, Coutts JR (1990). Induction of multiple follicular development for IVF. Br Med Bull.

[9] Mansour RT, Aboulghar MA, Serour GI (1994). Study of the optimum time for human chorionic gonadotropin-ovum pickup interval in in vitro fertilization. J Assist Reprod Genet.

[10] Wang W, Zhang XH, Wang WH, Liu YL, Zhao LH, Xue SL, Yang KH (2011). The time interval between hCG priming and oocyte retrieval in ART program: a meta-analysis. J Assist Reprod Genet.

[11] Bosdou JK, Kolibianakis EM, Venetis CA, Zepiridis L, Chatzimeletiou K, Makedos A, Triantafyllidis S, Masouridou S, Mitsoli A, Tarlatzis B (2015). Is the time interval between HCG administration and oocyte retrieval associated with oocyte retrieval rate?. Reprod Biomed Online.

[12] Shen X, Long H, Guo W, Xie Y, Gao H, Zhang J, Wang Y, Lyu Q, Kuang Y, Wang L (2020). The ovulation trigger-OPU time interval of different ovarian protocols in ART: a retrospective study. Arch Gynecol Obstet.

[13] Nargund G, Reid F, Parsons J (2001). Human chorionic gonadotropin-to-oocyte collection interval in a superovulation IVF program A prospective study. J Assist Reprod Genet.

[14] Shamseer L, Moher D, Clarke M, Ghersi D, Liberati A, Petticrew M, Shekelle P, Stewart LA, Group P-P (2015). Preferred reporting items for systematic review and meta-analysis protocols (PRISMA-P) 2015: elaboration and explanation. BMJ.

[15] Moher D, Shamseer L, Clarke M, Ghersi D, Liberati A, Petticrew M, Shekelle P, Stewart LA, Group P-P (2015). Preferred reporting items for systematic review and meta-analysis protocols (PRISMA-P) 2015 statement. Syst Rev.

[16] Sterne JAC, Savovic J, Page MJ, Elbers RG, Blencowe NS, Boutron I, Cates CJ, Cheng HY, Corbett MS, Eldridge SM (2019). RoB 2: a revised tool for assessing risk of bias in randomised trials. BMJ.

[17] Lo CK, Mertz D, Loeb M (2014). Newcastle-Ottawa Scale: comparing reviewers' to authors' assessments. BMC Med Res Methodol.

[18] Thornton SJ, Pantos C, Speirs A, Johnston I (1990). Human chorionic gonadotropin to oocyte retrieval interval in in vitro fertilization–how critical is it ?. Fertil Steril.

[19] Jamieson ME, Fleming R, Kader S, Ross KS, Yates RW, Coutts JR (1991). In vivo and in vitro maturation of human oocytes: effects on embryo development and polyspermic fertilization. Fertil Steril.

[20] Bjercke S, Tanbo T, Dale PO, Abyholm T (2000). Comparison between two hCG-to-oocyte aspiration intervals on the outcome of in vitro fertilization. J Assist Reprod Genet.

[21] Bokal EV, Vrtovec HM, Virant Klun I, Verdenik I (2005). Prolonged HCG action affects angiogenic substances and improves follicular maturation, oocyte quality and fertilization competence in patients with polycystic ovarian syndrome. Hum Reprod.

[22] Raziel A, Schachter M, Strassburger D, Kasterstein E, Ron-El R, Friedler S (2006). In vivo maturation of oocytes by extending the interval between human chorionic gonadotropin administration and oocyte retrieval. Fertil Steril.

[23] Gong SQ (2012). Effect of different timing of egg retrieval after HCG on IVF-ET outcomes. Mod Hosp.

[24] Ghasemian F, Faraji R, Asgharnia M, Zahiri Z, Bahadori MH (2013). The impact of different time intervals between hCG priming and oocyte retrieval on ART outcomes. Iran J Reprod Med.

[25] Chen YY, Liu LL, Cai JL, Li P, Ren JZ (2014). SHa AG: Comparison among hCG-to-oocyte aspiration intervals on quality of ovum and embryo. Clin J Med Offic.

[26] Bao LL, Wu XH, Wang DX, Liu SH (2019). The effect of prolonged HCG exposure time during IVF on the clinical outcome of PCOS. Prog Obstet Gynecol.

[27] Skvirsky S, Blais I, Lahav-Baratz S, Koifman M, Wiener-Megnazi Z, Dirnfeld M (2021). Time interval between hCG administration and oocyte pick up: analysis of oocyte maturation, embryonic morphology, morphokinetics, and IVF outcome. Clin Exp Obstet Gynecol.

[28] Wang X, Xiao Y, Sun Z, Zhen J, Yu Q (2021). Effect of the time interval between oocyte retrieval and ICSI on embryo development and reproductive outcomes: a systematic review. Reprod Biol Endocrinol.

[29] Schmidt DW, Maier DB, Nulsen JC, Benadiva CA (2004). Reducing the dose of human chorionic gonadotropin in high responders does not affect the outcomes of in vitro fertilization. Fertil Steril.

[30] Isik AZ, Vicdan K (2001). Combined approach as an effective method in the prevention of severe ovarian hyperstimulation syndrome. Eur J Obstet Gynecol Reprod Biol.

[31] Alper MM, Fauser BC (2017). Ovarian stimulation protocols for IVF: is more better than less?. Reprod Biomed Online.

[32] Son WY, Chung JT, Chian RC, Herrero B, Demirtas E, Elizur S, Gidoni Y, Sylvestre C, Dean N, Tan SL (2008). A 38 h interval between hCG priming and oocyte retrieval increases in vivo and in vitro oocyte maturation rate in programmed IVM cycles. Hum Reprod.

[33] Weiss A, Neril R, Geslevich J, Lavee M, Beck-Fruchter R, Golan J, Shalev E (2014). Lag time from ovulation trigger to oocyte aspiration and oocyte maturity in assisted reproductive technology cycles: a retrospective study. Fertil Steril.

[34] Deng M, Liang Y, Qin H, Tan Y, Mai Q, Yuan X, Yuan Y (2020). A moderately extended time interval between hCG administration and oocyte retrieval is good for most patients with oocyte retrieval scheduled on the same day: a retrospective cohort study. J Obstet Gynaecol.

[35] Garor R, Shufaro Y, Kotler N, Shefer D, Krasilnikov N, Ben-Haroush A, Pinkas H, Fisch B, Sapir O (2015). Prolonging oocyte in vitro culture and handling time does not compensate for a shorter interval from human chorionic gonadotropin administration to oocyte pickup. Fertil Steril.

[36] Geng Y, Lai Q, Xun Y, Jin L (2018). The effect of premature luteinizing hormone increases among high ovarian responders undergoing a gonadotropin-releasing hormone antagonist ovarian stimulation protocol. Int J Gynaecol Obstet.

[37] Al Rahwanji MJ, Abouras H, Shammout MS, Altalla R, Al Sakaan R, Alhalabi N, Alhalabi M (2022). The optimal period for oocyte retrieval after the administration of recombinant human chorionic gonadotropin in in vitro fertilization. BMC Pregnancy Childbirth.

[38] Vandenberghe LTM, Santos-Ribeiro S, De Munck N, Desmet B, Meul W, De Vos A, Van de Velde H, Racca A, Tournaye H, Verheyen G (2021). Expanding the time interval between ovulation triggering and oocyte injection: does it affect the embryological and clinical outcome?. Hum Reprod.

[39] Eppig JJ, Schultz RM, O'Brien M, Chesnel F (1994). Relationship between the developmental programs controlling nuclear and cytoplasmic maturation of mouse oocytes. Dev Biol.

[40] Van de Velde H, De Vos A, Joris H, Nagy ZP, Van Steirteghem AC (1998). Effect of timing of oocyte denudation and micro-injection on survival, fertilization and embryo quality after intracytoplasmic sperm injection. Hum Reprod.

[41] Ho JY, Chen MJ, Yi YC, Guu HF, Ho ES (2003). The effect of preincubation period of oocytes on nuclear maturity, fertilization rate, embryo quality, and pregnancy outcome in IVF and ICSI. J Assist Reprod Genet.

[42] Pereira N, Neri QV, Lekovich JP, Palermo GD, Rosenwaks Z (2016). The role of in-vivo and in-vitro maturation time on ooplasmic dysmaturity. Reprod Biomed Online.

[43] Alvarez Sedo C, Miguens M, Andreucci S, Ortiz N, Lorenzi D, Papier S, Nodar F (2015). Correlation between Cytoplamic Oocyte Maturation and Chromosomal Aneuploidies - Impact on fertilization, embryo quality and pregnancy. JBRA Assist Reprod.

[44] Kuo TC, Endo K, Dharmarajan AM, Miyazaki T, Atlas SJ, Wallach EE (1991). Direct effect of angiotensin II on in-vitro perfused rabbit ovary. J Reprod Fertil.

[45] Artini PG, Fasciani A, Monti M, Luisi S, D'Ambrogio G, Genazzani AR (1998). Changes in vascular endothelial growth factor levels and the risk of ovarian hyperstimulation syndrome in women enrolled in an in vitro fertilization program. Fertil Steril.

[46] Templeton AA, van Look P, Angell RE, Aitken RJ, Lumsden MA, Baird DT (1986). Oocyte recovery and fertilization rates in women at various times after the administration of hCG. J Reprod Fertil.

[47] Andersen AG, Als-Nielsen B, Hornnes PJ, Franch Andersen L (1995). Time interval from human chorionic gonadotrophin (HCG) injection to follicular rupture. Hum Reprod.

