# Supplementary figures and images for: Time interval between hCG administration and oocyte retrieval and ART outcomes: an updated systematic review and meta-analysis
Source: Reprod Biol Endocrinol. 2023 Jul 3;21:61. doi: 10.1186/s12958-023-01110-9 (PMC10316642; doi:10.1186/s12958-023-01110-9)

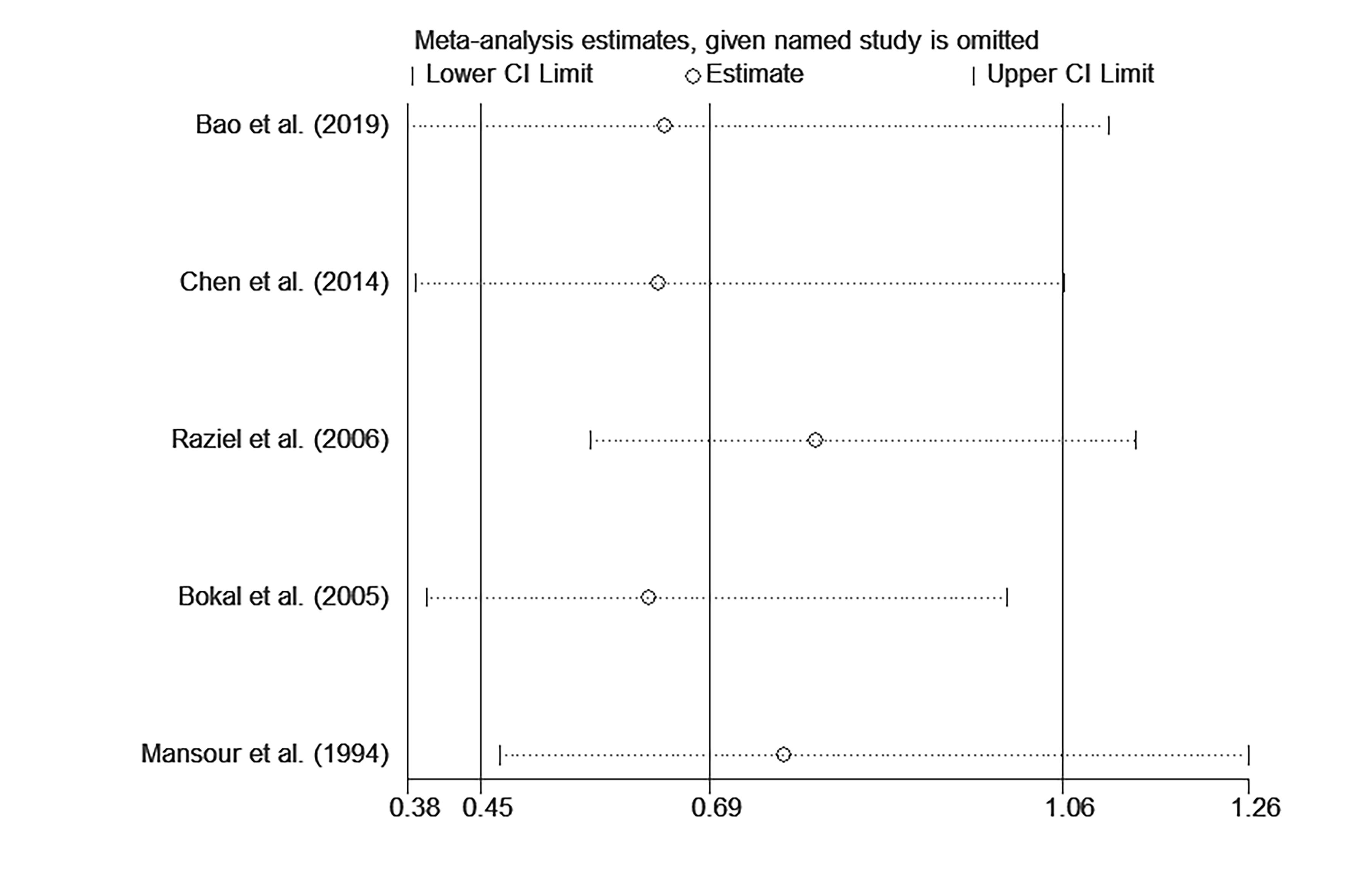

Supplement: Supplementary file 1 — Additional file 1: Figure S1. Meta-analysis, sensitivity analysis, and random-effects estimates examining the oocyte maturation rate of short interval versus long interval in ART program. [file 12958_2023_1110_MOESM1_ESM.jpg]

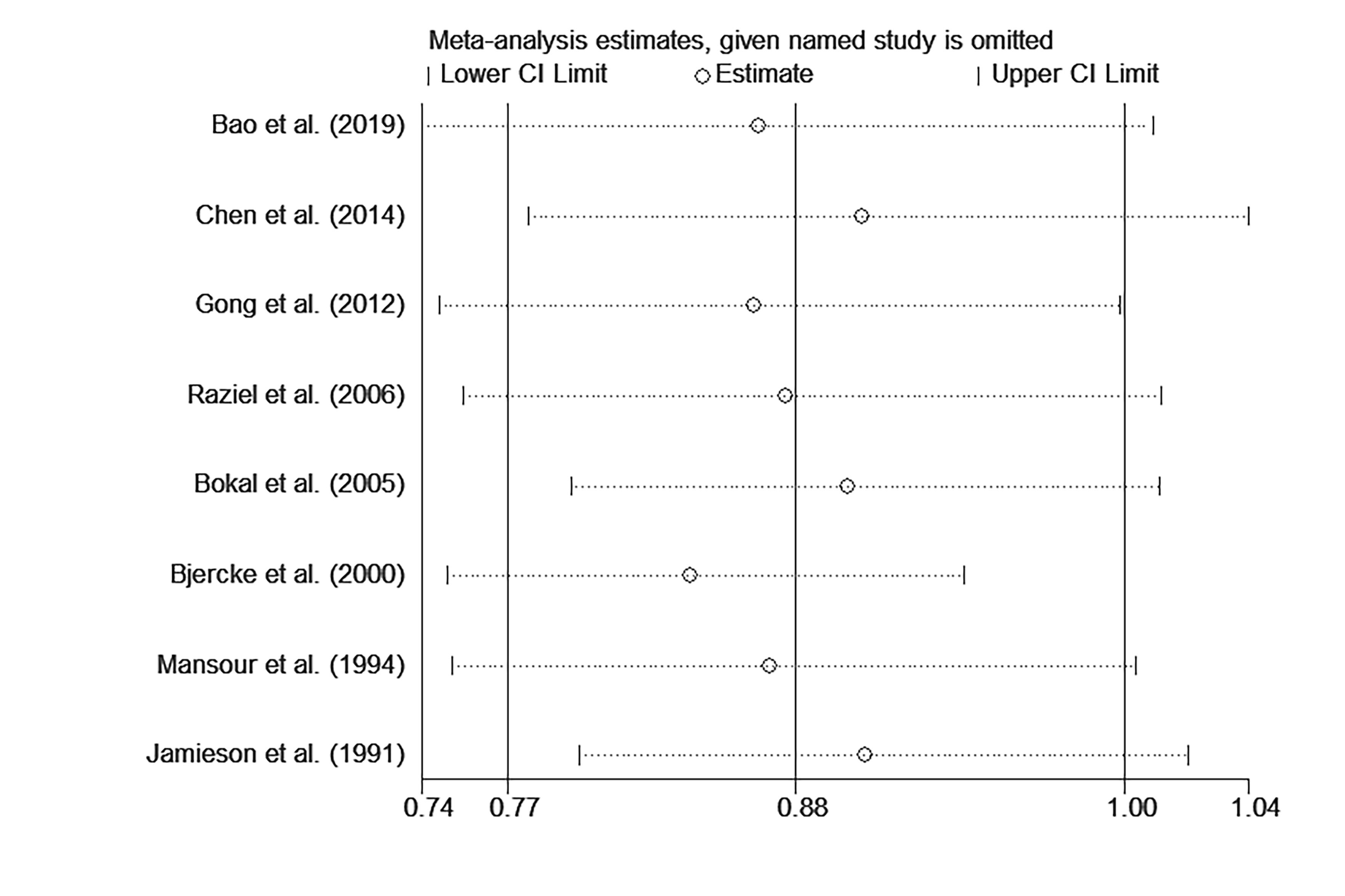

Supplement: Supplementary file 2 — Additional file 2: Figure S2. Meta-analysis, sensitivity analysis, and random-effects estimates examining the fertilization rate of short interval versus long interval in ART program. [file 12958_2023_1110_MOESM2_ESM.jpg]

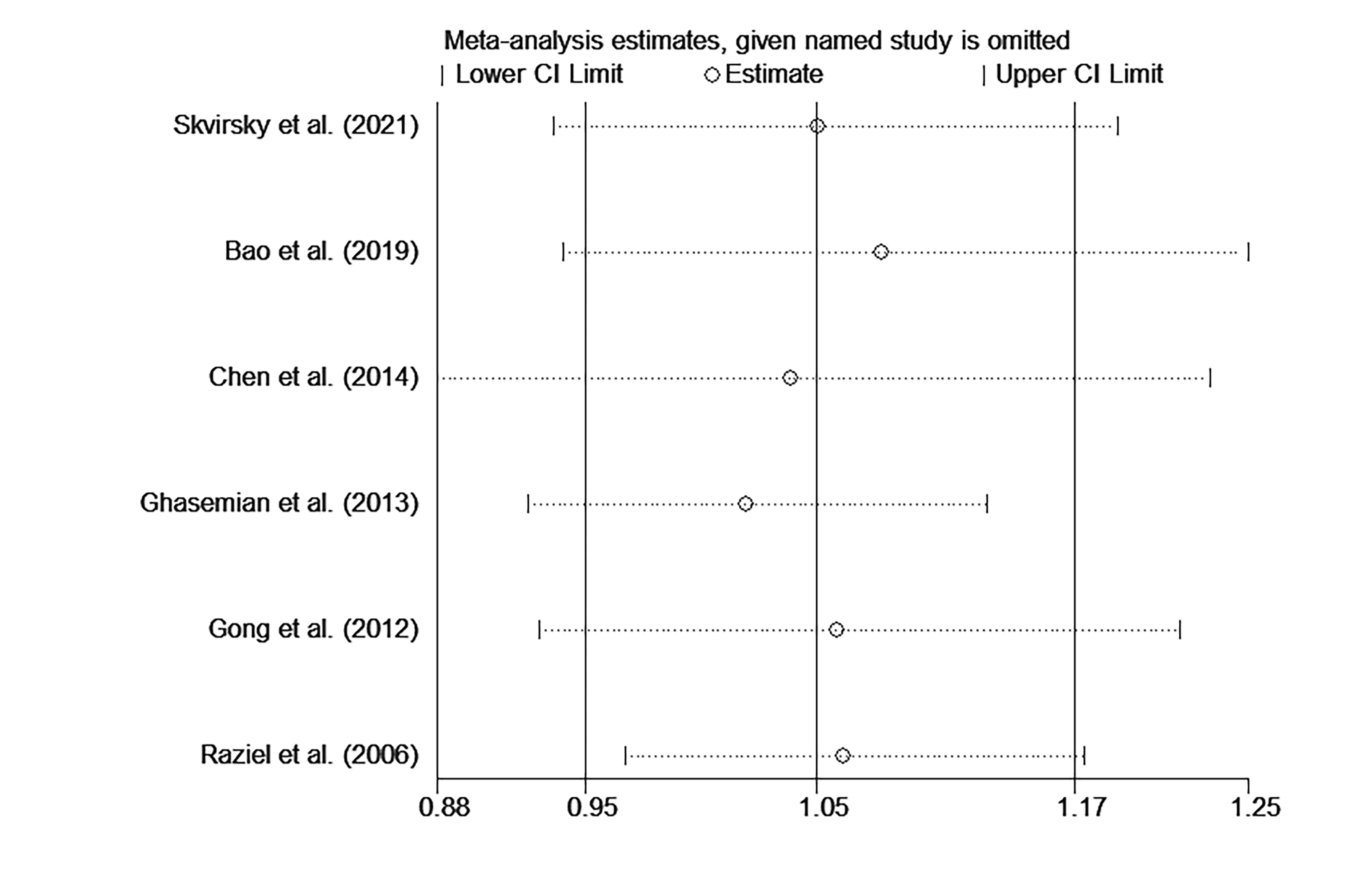

Supplement: Supplementary file 3 — Additional file 3: Figure S3. Meta-analysis, sensitivity analysis, and random-effects estimates examining the high-quality rate of short interval versus long interval in ART program. [file 12958_2023_1110_MOESM3_ESM.tif]

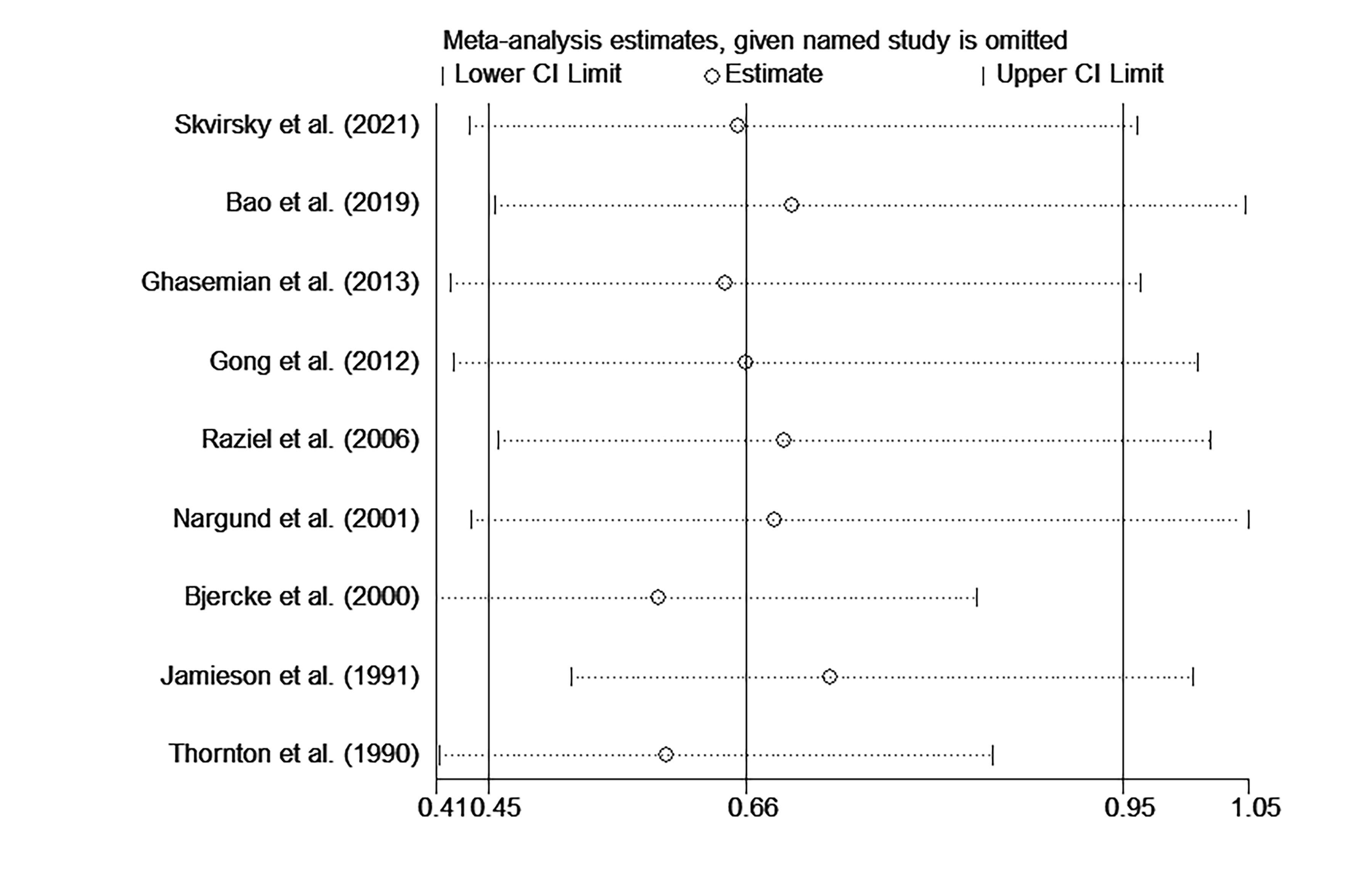

Supplement: Supplementary file 4 — Additional file 4: Figure S4. Meta-analysis, sensitivity analysis, and random-effects estimates examining the clinical pregnancy rate of short interval versus long interval in ART program. [file 12958_2023_1110_MOESM4_ESM.jpg]

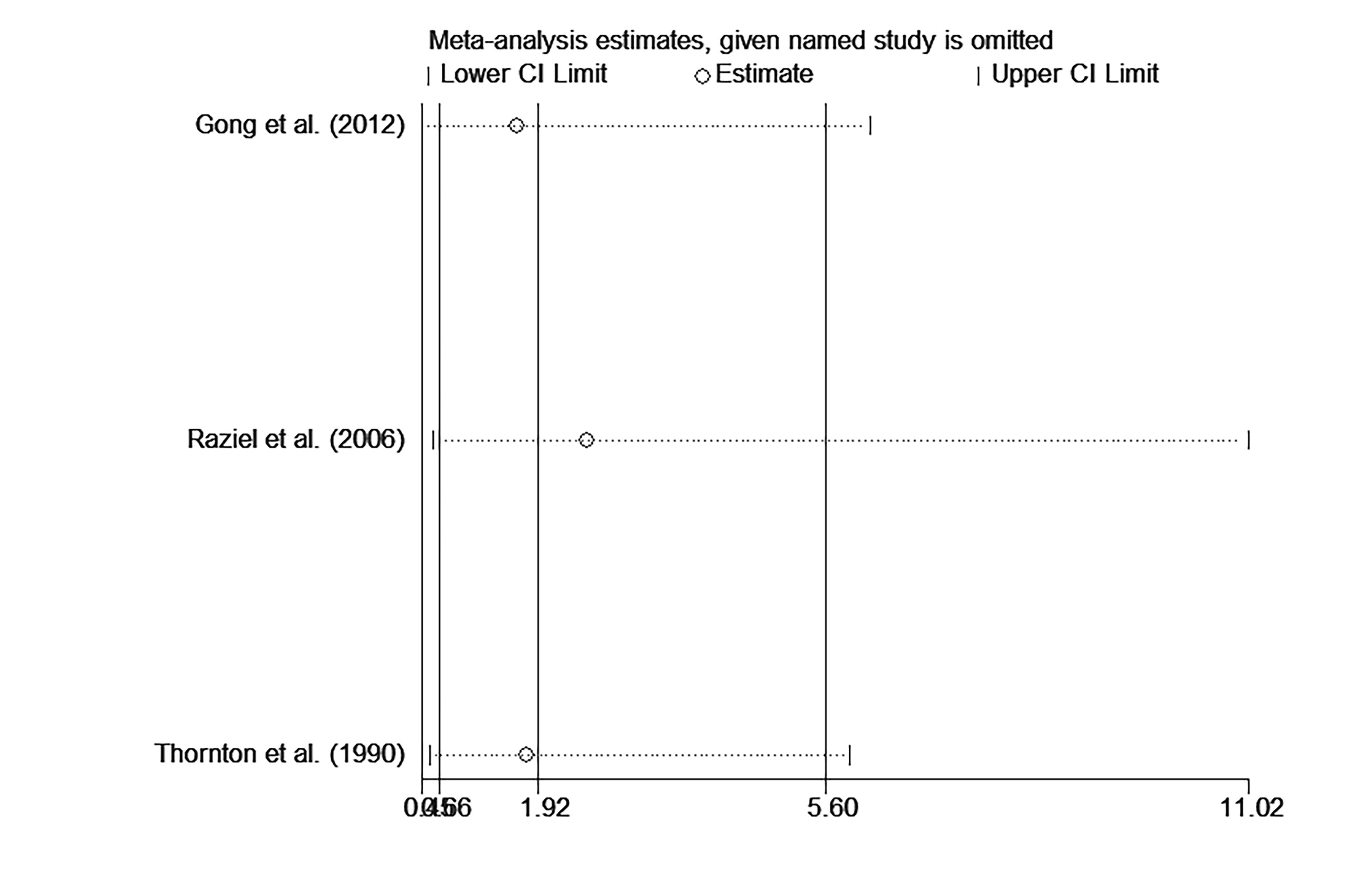

Supplement: Supplementary file 5 — Additional file 5: Figure S5. Meta-analysis, sensitivity analysis, and random-effects estimates examining the miscarriage rate of short interval versus long interval in ART program. [file 12958_2023_1110_MOESM5_ESM.jpg]

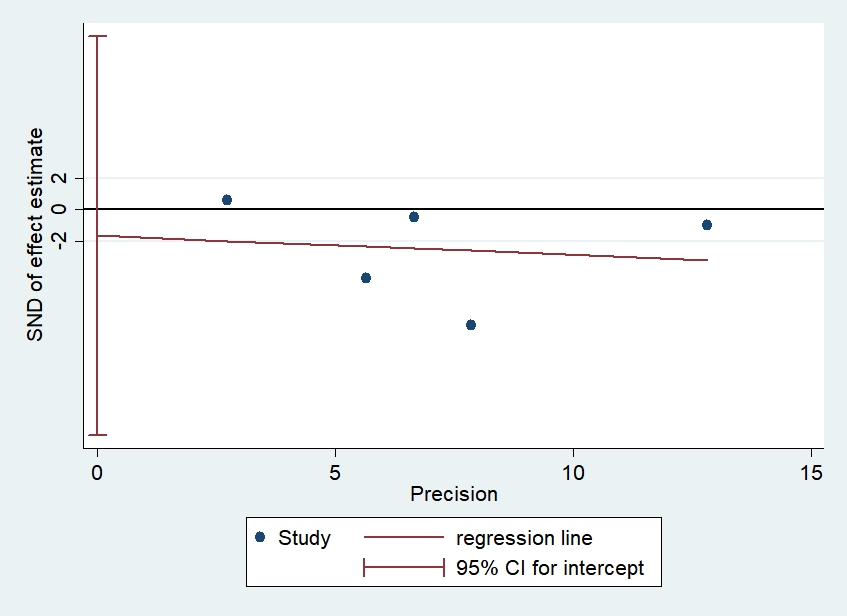

Supplement: Supplementary file 6 — Additional file 6: Figure S6. Publication bias analysis of oocyte maturation rate of short interval versus long interval in ART program. [file 12958_2023_1110_MOESM6_ESM.jpg]

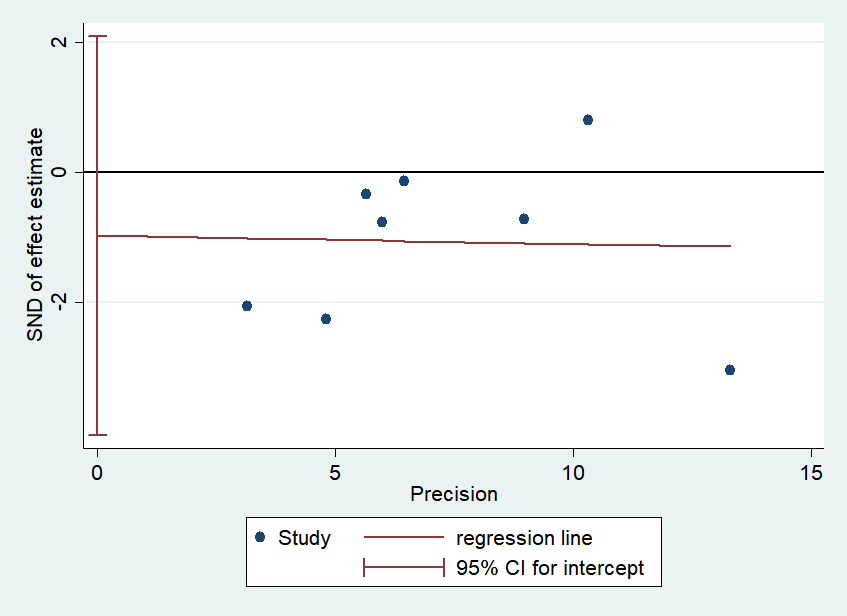

Supplement: Supplementary file 7 — Additional file 7: Figure S7. Publication bias analysis of fertilization rate of short interval versus long interval in ART program. [file 12958_2023_1110_MOESM7_ESM.tif]

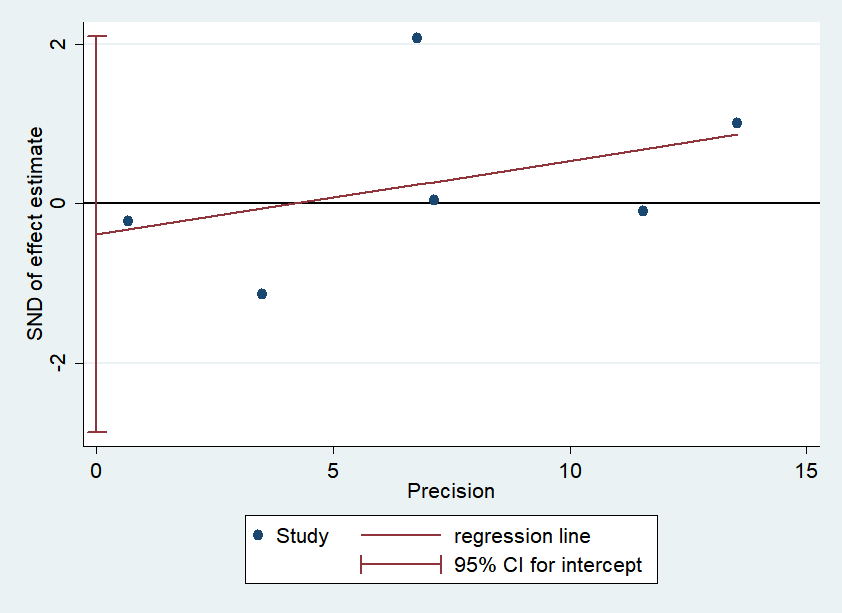

Supplement: Supplementary file 8 — Additional file 8: Figure S8. Publication bias analysis of high-quality rate of short interval versus long interval in ART program. [file 12958_2023_1110_MOESM8_ESM.tif]

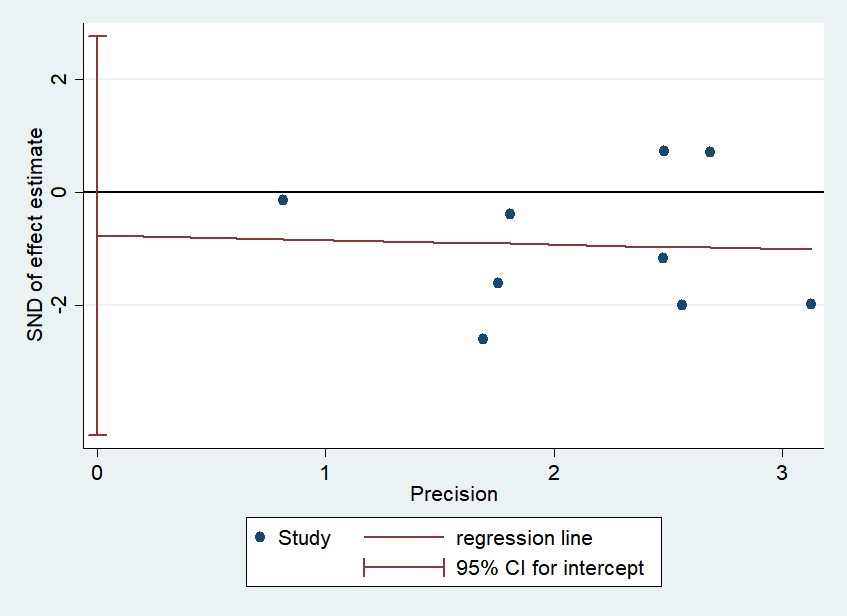

Supplement: Supplementary file 9 — Additional file 9: Figure S9. Publication bias analysis of clinical pregnancy rate of short interval versus long interval in ART program. [file 12958_2023_1110_MOESM9_ESM.tif]

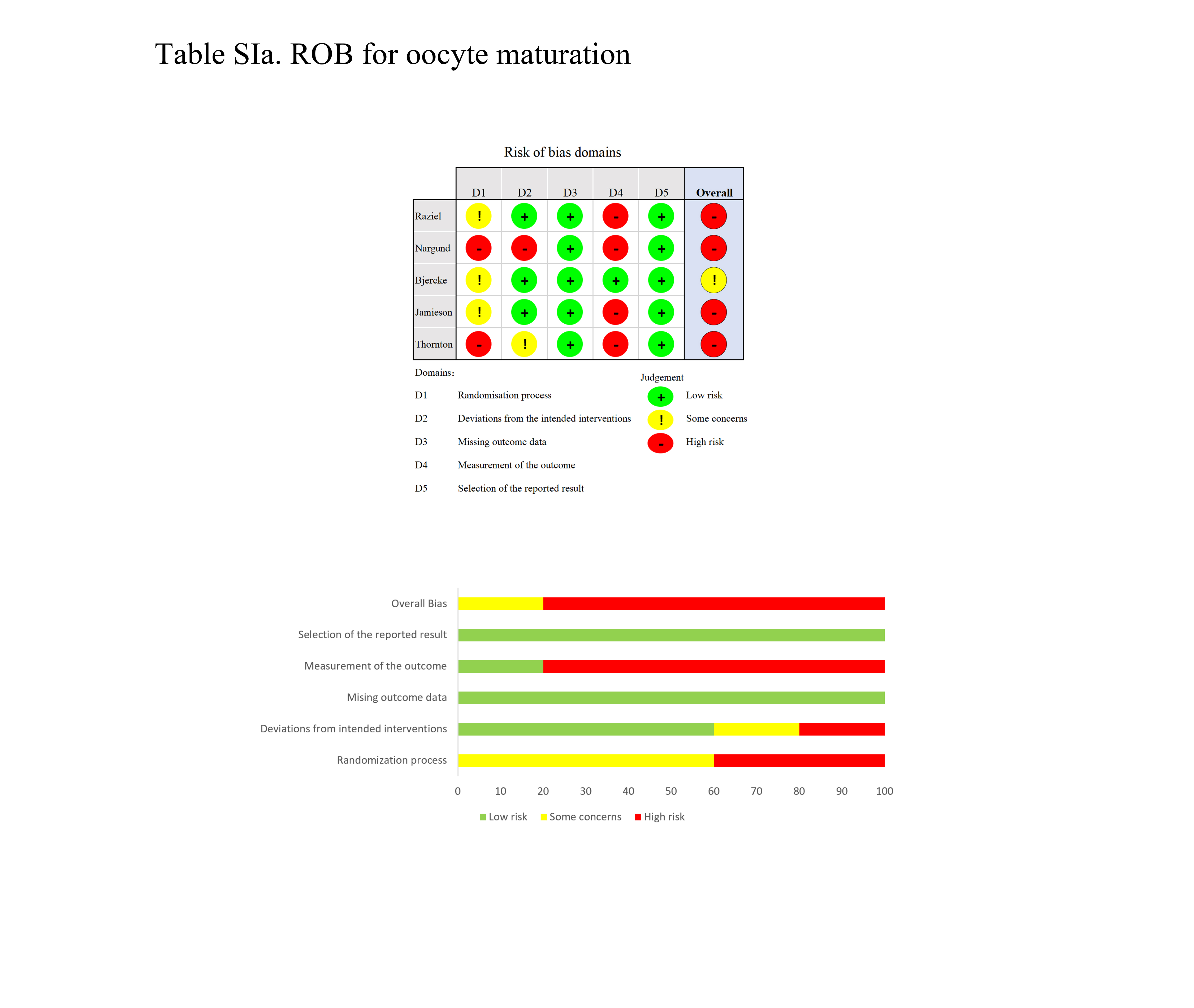

Supplement: Supplementary file 10 — Additional file 10: Table SIa. ROB for oocyte maturation outcome. Table SIb. ROB for fertilization outcome. Table SIc. ROB for high-quality embryo outcome. Table SId. ROB for clinical pregnancy outcome. Table SIe. ROB for miscarriage outcome. [file 12958_2023_1110_MOESM10_ESM.zip › Table SIa.jpg]

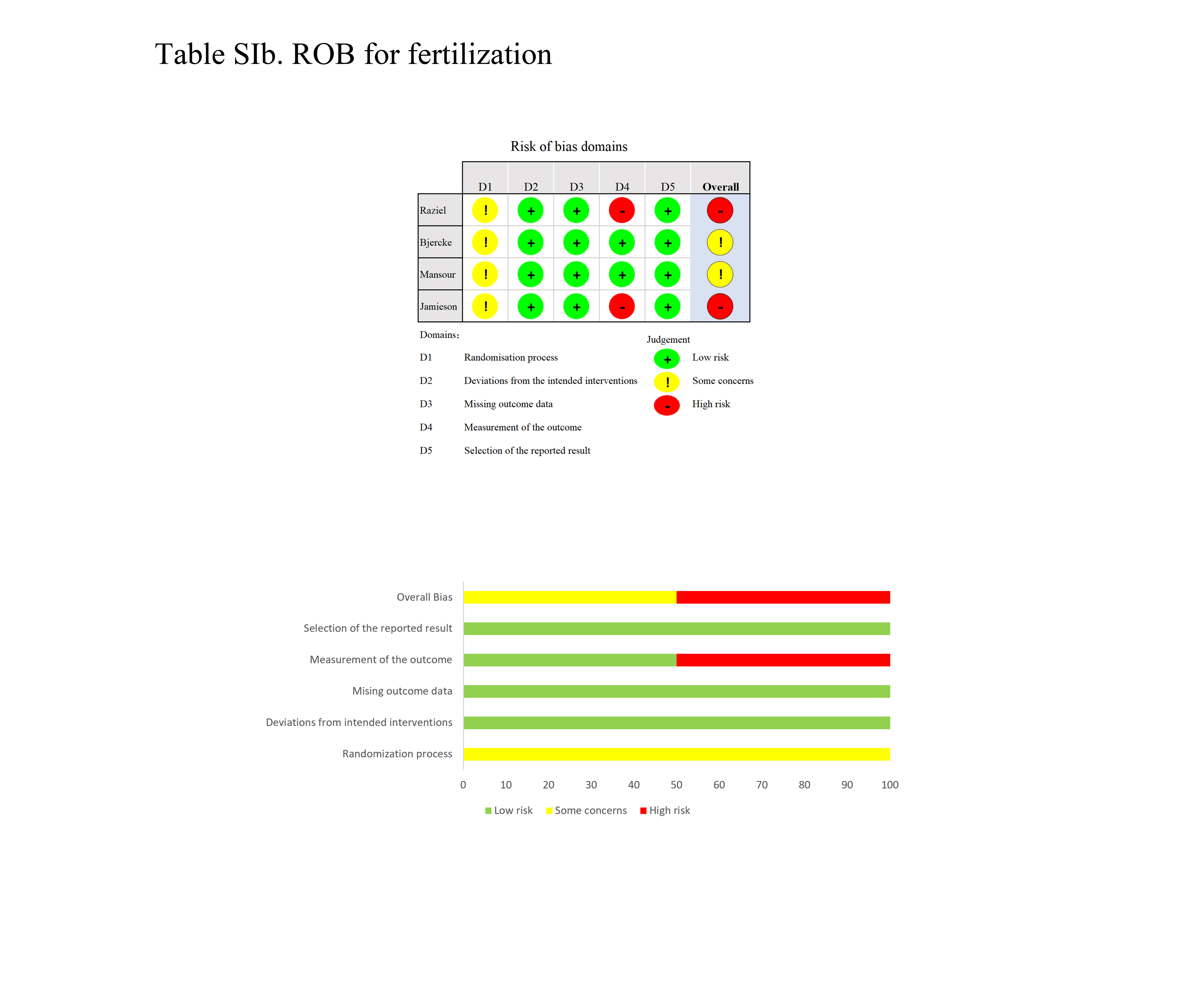

Supplement: Supplementary file 10 — Additional file 10: Table SIa. ROB for oocyte maturation outcome. Table SIb. ROB for fertilization outcome. Table SIc. ROB for high-quality embryo outcome. Table SId. ROB for clinical pregnancy outcome. Table SIe. ROB for miscarriage outcome. [file 12958_2023_1110_MOESM10_ESM.zip › Table SIb.jpg]

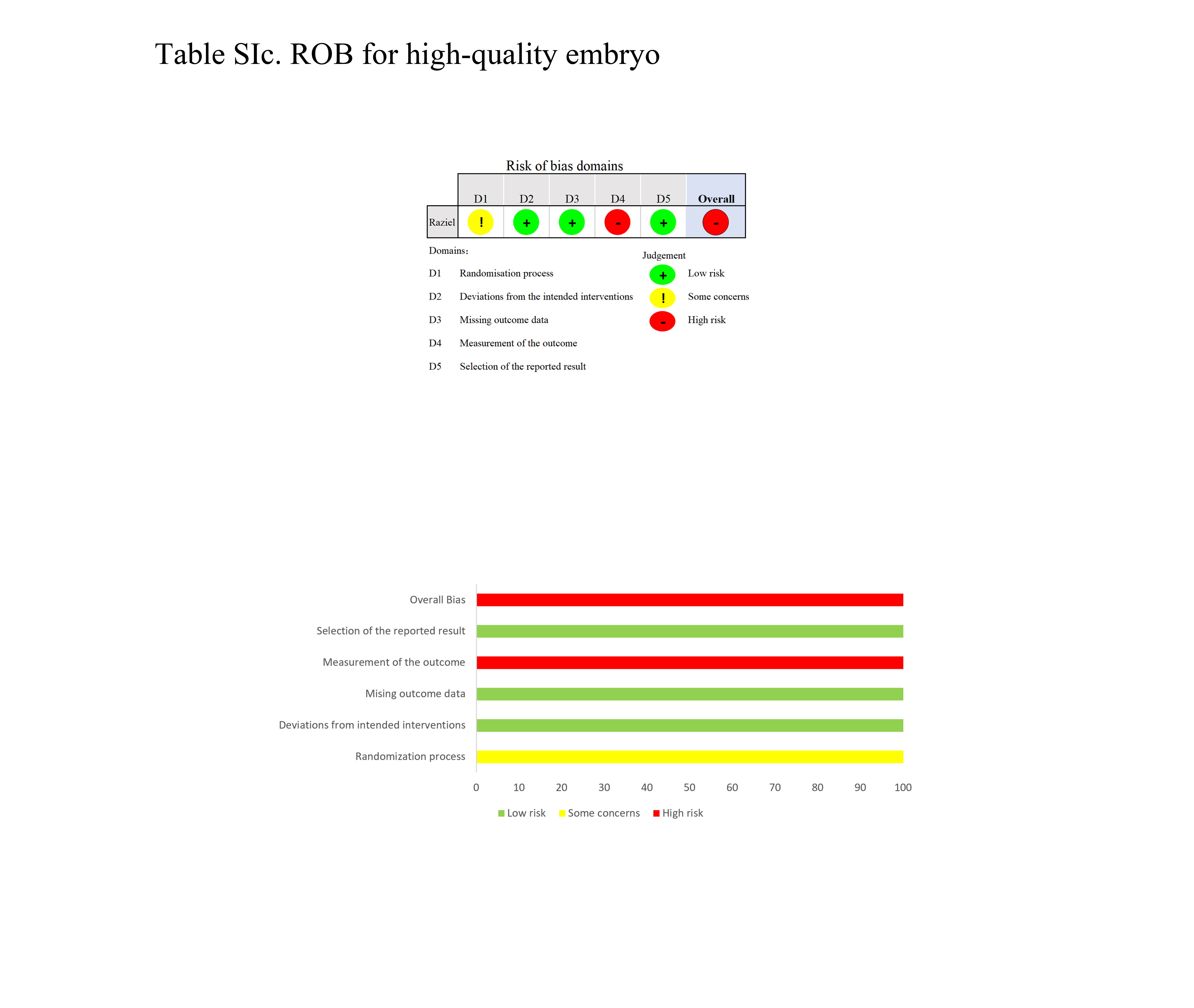

Supplement: Supplementary file 10 — Additional file 10: Table SIa. ROB for oocyte maturation outcome. Table SIb. ROB for fertilization outcome. Table SIc. ROB for high-quality embryo outcome. Table SId. ROB for clinical pregnancy outcome. Table SIe. ROB for miscarriage outcome. [file 12958_2023_1110_MOESM10_ESM.zip › Table SIc.jpg]

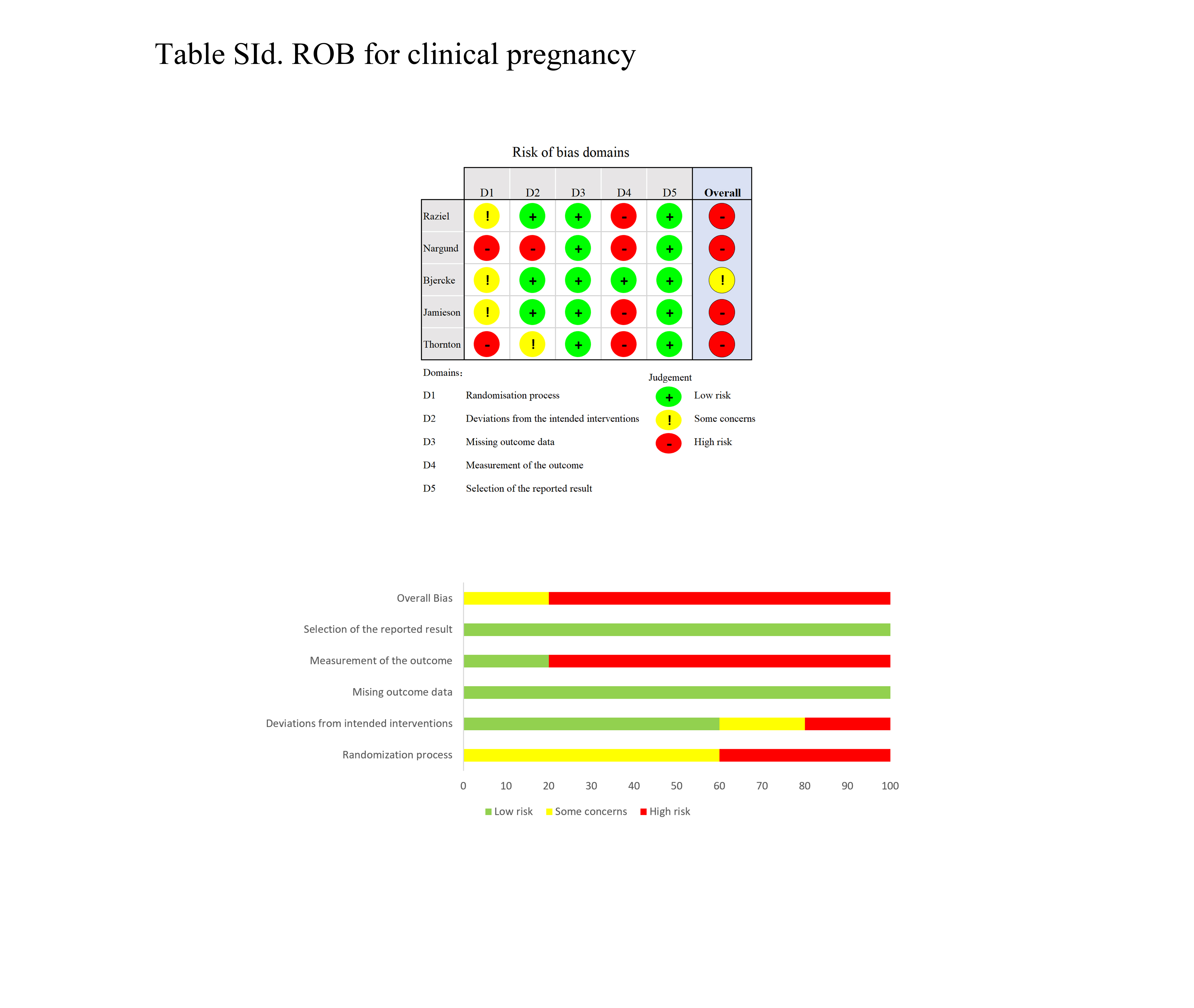

Supplement: Supplementary file 10 — Additional file 10: Table SIa. ROB for oocyte maturation outcome. Table SIb. ROB for fertilization outcome. Table SIc. ROB for high-quality embryo outcome. Table SId. ROB for clinical pregnancy outcome. Table SIe. ROB for miscarriage outcome. [file 12958_2023_1110_MOESM10_ESM.zip › Table SId.jpg]

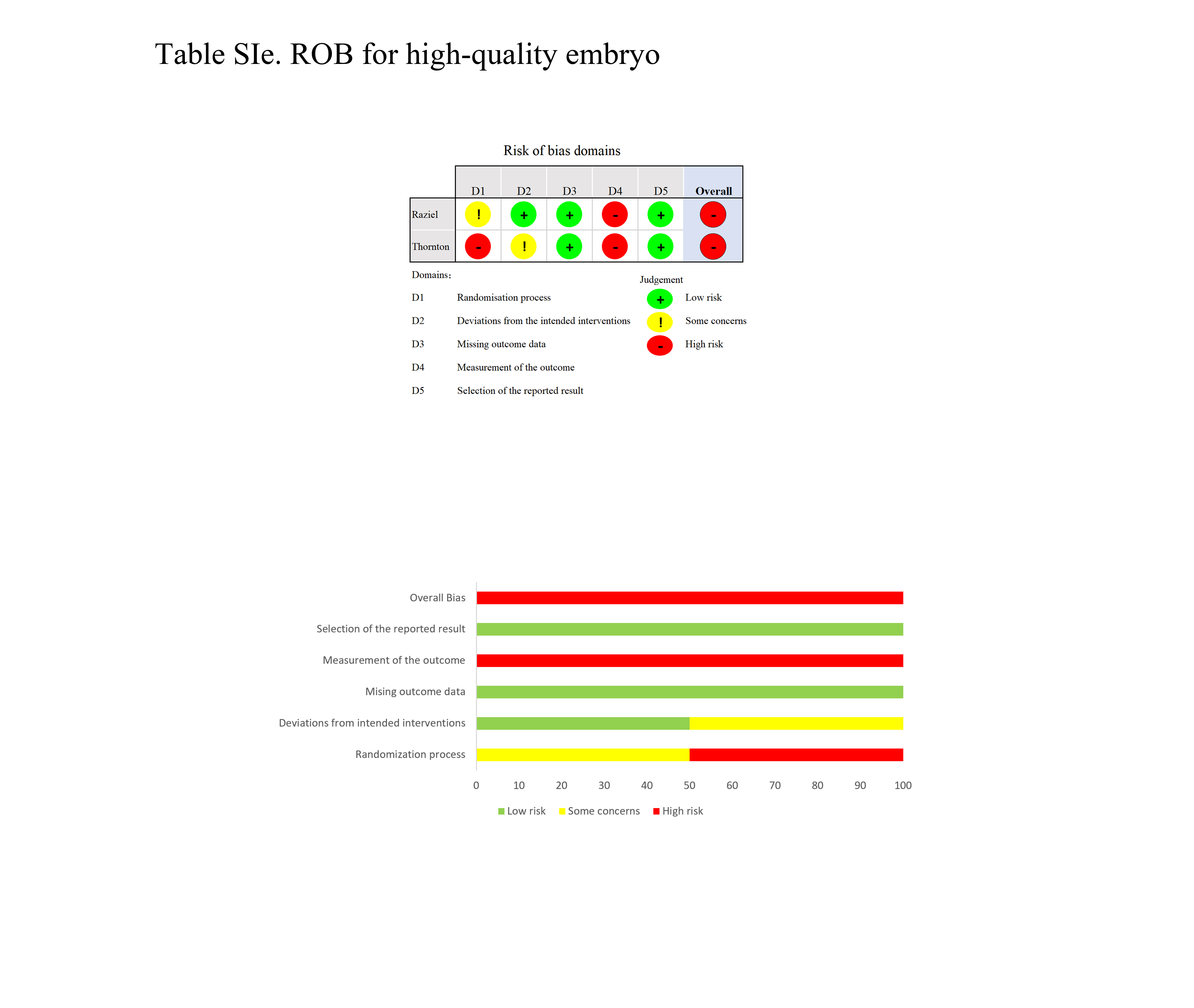

Supplement: Supplementary file 10 — Additional file 10: Table SIa. ROB for oocyte maturation outcome. Table SIb. ROB for fertilization outcome. Table SIc. ROB for high-quality embryo outcome. Table SId. ROB for clinical pregnancy outcome. Table SIe. ROB for miscarriage outcome. [file 12958_2023_1110_MOESM10_ESM.zip › Table SIe.jpg]
